# Healthcare costs and medical utilization patterns associated with painful and severe painful diabetic peripheral neuropathy

**DOI:** 10.1007/s12020-024-03954-6

**Published:** 2024-07-13

**Authors:** Todd Bromberg, Nicolas C. Gasquet, Christine N. Ricker, Charlotte Wu

**Affiliations:** 1 Delaware Valley Pain & Spine Institute, Chalfont, PA USA; 2 Medtronic Plc, Minneapolis, MN USA

**Keywords:** Peripheral neuropathy, Pain, Type 1 diabetes, Type 2 diabetes, Medical resource use, Costs

## Abstract

**Purpose:**

Painful diabetic peripheral neuropathy (DPN) is a common complication in patients with diabetes. It is associated with a poor quality of life and high costs of care. This study investigated the impact of painful DPN on healthcare costs and resource utilization.

**Methods:**

This was a retrospective analysis of administrative claims of adult patients with diabetes (type 1 or 2) from Optum’s de-identified Clinformatics® Data Mart Database. Patients were assigned to four cohorts by presence of DPN and pain severity, based on diagnoses and prescription patterns in a one-year baseline. All-cause and diabetes-associated costs were calculated for the year following the index DPN diagnosis. Risk factors associated with presence of severely painful DPN were evaluated.

**Results:**

Relative to those without DPN, patients who had DPN without pain, painful DPN (PDPN), or severe PDPN incurred respective increases of $3,093, $9,349, and $20,887 in average annual all-cause costs. More than half of costs from painful/severe DPN were for prescriptions and inpatient hospitalization. Severe PDPN was associated with elevated odds of diabetic amyotrophy (OR: 8.09; 95% CI: 6.84–9.56), diabetic foot ulcers (OR: 6.54, 95% CI: 6.32–6.76), and loss of mobility (OR: 2.54, 95% CI: 2.48–2.60), among other complications.

**Conclusions:**

Painful DPN is associated with higher healthcare costs and resource utilization, and a greater risk of debilitating conditions that limit quality of life. Future research should focus on better treatment options and more aggressive pain management strategies to reduce the negative impacts of DPN.

## Introduction

Diabetes prevalence rates have been increasing in recent years, making it one of the most common diseases in the world. In 2019 in the United States, it was estimated that ~37.3 million people had some form of diabetes (diabetes type 1, 2, or gestational), or 14.7% of the entire US adult population [1]. If left untreated or undertreated, diabetes can lead to many complications, including increased risk of heart attack and stroke, peripheral vascular disease, kidney damage, retinopathy, and neuropathy. Diabetic peripheral neuropathy (DPN) is a common complication with a prevalence ranging between 6 and 51%. In adult patients with type 1 diabetes, prevalence ranged from 18% (18- to 29-year-olds) to 58% (30 years of age or older), and among adult patients with type 2 diabetes it ranges from 39% to 51%. The risk of developing DPN increases with age and is expected to occur in 50% of patients with diabetes over their lifetime [2].

The prevalence of painful diabetic peripheral neuropathy (PDPN), with symptoms such as burning sensations and stabbing-like pain in the lower extremities, is estimated to range between 10 and 30% among those with diabetes alone and 40%–50% among those with DPN [2, 3]. PDPN is associated with a worse quality of life due to symptoms such as burning or tingling pain, electric pain, and pain while walking [4, 5], as well as reduced quality of sleep, activity, and productivity [6]. Additionally, management of patients with PDPN places a high financial burden on health plans. In a prior retrospective claims analysis, patients with diabetes alone incurred total annual medical costs to their health plan of $6,632, whereas patients with diabetic complications, including PDPN, had much higher annual costs ranging from $12,492 to $30,655 [7].

Evidence regarding the impact of DPN pain severity on total cost burden remains limited. One prior study used a combination of diagnosis codes and pain scores from electronic health records to categorize patients with different PDPN severity levels and reported associated costs [6]. However, this appears to be the only study to date that has quantified the costs of painful and severe PDPN, and the cost information is only current as of 2013.

The objective of the present study was to retrospectively assess payer and out-of-pocket patient costs associated with management of DPN, using contemporary cost information. A secondary objective was to explore risk factors correlated with presence of severe PDPN.

## Materials and methods

### Data sources

Optum’s de-identified Clinformatics® Data Mart Database of administrative claims data was used as the source of data for this study. This database consists of administrative health claims for members of large commercial and Medicare Advantage health plans. Data in the Clinformatics® Data Mart are de-identified in accordance with the HIPAA Privacy Rule, and this study was determined exempt from full review by Sterling IRB (Atlanta, GA).

### Study timeline and definitions

**Fig. 1:**
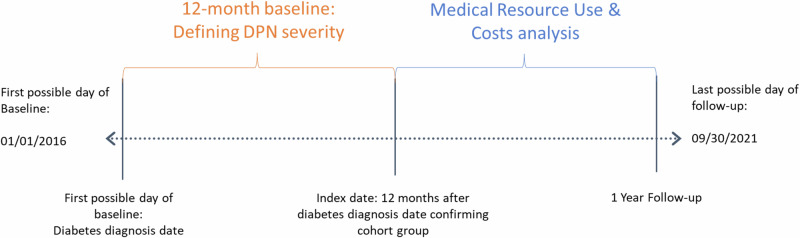
Study timeline

To evaluate patient eligibility for the study, an identification window between January 1 of 2016, and September 30 of 2020 was reviewed to identify patients with a diagnosis of either type 1 or 2 diabetes (Fig. 1). The date of first observed diabetes diagnosis was designated as the “diabetes diagnosis date”. A 12-month baseline period following the diabetes diagnosis date was used to stratify patients into four cohorts according to degree of DPN present—no DPN, DPN without pain, PDPN, and severe PDPN. The last date of baseline was considered the index date. Data from a one-year follow-up period following the index date was used to assess differences in medical resource use and costs between the four cohorts.

### Patient selection and cohort assignment

**Fig. 2:**
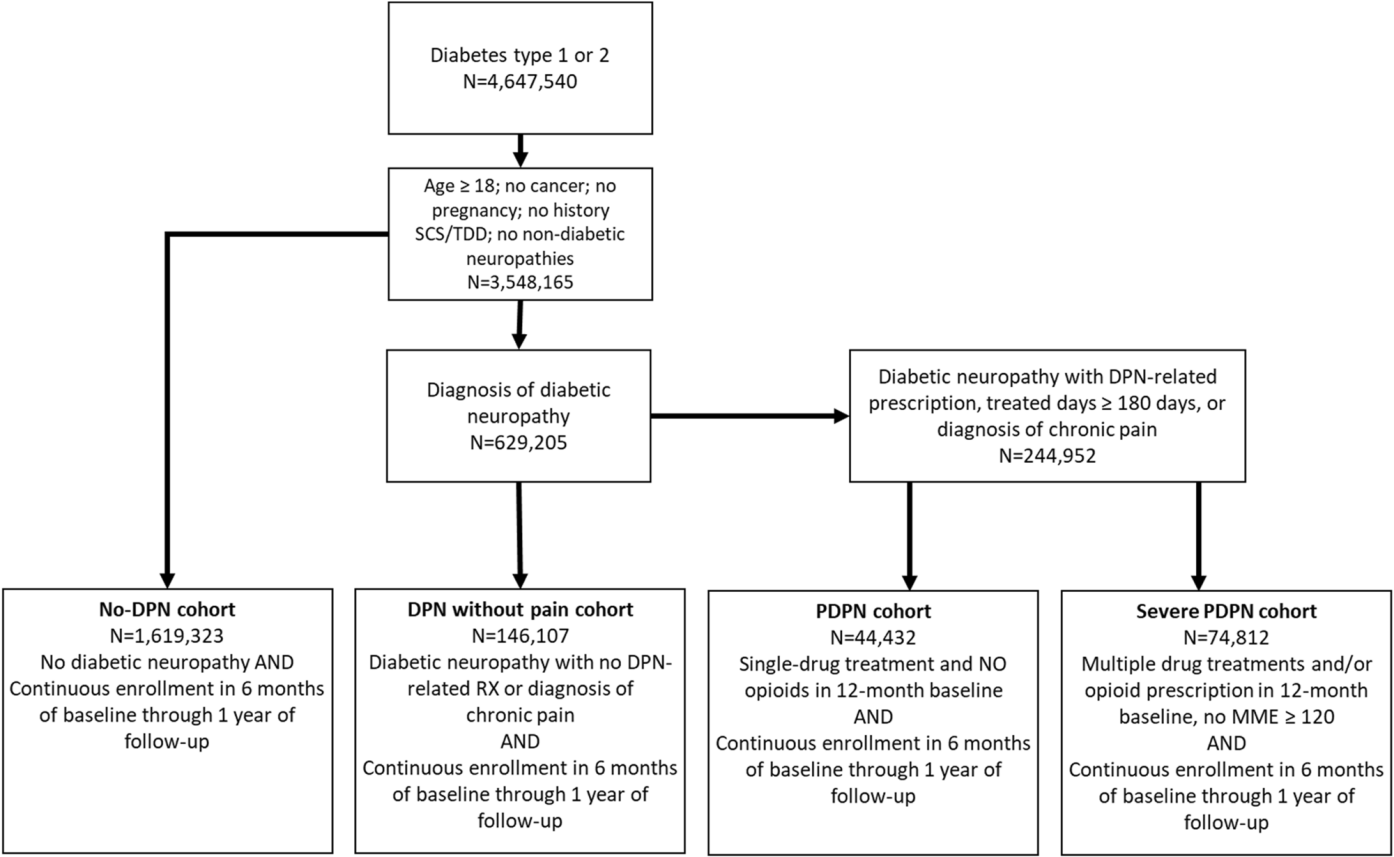
Cohort structure and patient selection

To be eligible for the study, all patients were required to have at least a primary or secondary diagnosis of type 1 or type 2 diabetes between January 2016 and September 2020 (Fig. 2). Patients without DPN were assigned to the no-DPN cohort. Patients in the DPN without pain cohort were required to have, at minimum, a primary or secondary diagnosis of DPN, but no prescription medication claims related to DPN or diagnosis of chronic pain in baseline. Medications related to treatment of nerve-related pain evaluated included amitriptyline, capsaicin, duloxetine, gabapentin, nortriptyline, opioids, pregabalin, sodium valproate anticonvulsants, and venlafaxine antidepressants.

The claims-based data source used for this analysis did not include pain score information, therefore we assigned patients to the PDPN and severe PDPN based upon diagnosis and intensity of diabetic neuropathy pain medication use. The PDPN cohort was required to have a DPN diagnosis AND at least one prescription fill of a diabetic neuropathy pain-related drug (excluding opioids) or a diagnosis of chronic pain. Patients defined as having severe PDPN were required to have a prescription fill of two or more classes of neuropathic pain medications used for control of DPN-related pain and at least one opioid. The rationale for separating PDPN from severe PDPN using these criteria was based on existing literature showing average prescription medication intensity is higher among those with severe pain [6]. We assigned patients with any opioid prescription fill to the severe PDPN group as opioids are not recommended as a first-line medication option for nerve-related pain.

Patients with prescription fills indicating an average daily morphine milligram equivalent of ≥120 mg/day were considered outliers and were excluded from the analyses. Patients were also excluded for diagnoses of non-diabetic neuropathies, diagnosis of cancer at any point in the study time period, pregnancy, a history of spinal cord stimulation or targeted drug delivery, and patients younger than 18. Continuous enrollment in a healthcare plan, from baseline through follow-up, was required for all cohorts.

### Study measures

Patient demographics and clinical characteristics were assessed in baseline. Demographics included age, race, geographic region, and sex. Clinical characteristics included the Charlson Comorbidity Index (CCI) score, the adapted Diabetes Complications Severity Index (aDCSI) [8], and diagnoses for anxiety, depression, loss of mobility, muscle weakness, diabetic foot ulcer and diabetic amyotrophy (Appendix 1, Table S1). Details of diabetes summarized included: among those with type 1 the proportion with use of continuous glucose monitoring (CGM), among those with type 2 the proportion with a prescription for insulin or a non-insulin antidiabetic medication (including Metformin, Sulfonylureas, Glinides, Thiazolidinediones, Acarbose, DPP-4 Inhibitors and GLP-1 analogs, Pramlintide, etc) and the number of treated days with the prescription in year one of follow-up.

Healthcare medical resource use and costs in follow-up were assessed both for all-cause visits not restricted to specific diagnoses and for those with a diagnosis of primary or secondary diabetes reported during the visit. Medical resource use was measured as the proportion of follow-up encounters by place of service, including inpatient hospital, outpatient hospital, ambulatory surgical centers, offices/clinics, emergency departments, and pharmacy prescriptions.

The costs were assessed as the sum of payer and patient out-of-pocket costs, which included deductibles, copayments, and coinsurance. Sensitivity analyses were performed to assess costs of the severe PDPN cohort compared to a composite of no-DPN, DPN without pain, and PDPN, as well as the costs of the PDPN cohort compared to costs of the combined no-DPN and DPN without pain cohorts. All costs were adjusted to 2021 USD.

### Analytical methods

Descriptive analysis used mean and standard deviations (SD) to report on continuous variables, and percentages were used to report on categorical variables. Chi-Square tests were used for categorical variables and one-way analysis of variance (ANOVA) tests for continuous variables to assess statistically significant differences between all four cohorts, as well as the sensitivity analyses. Three multivariate logistic regression models evaluated clinical characteristics correlated with severe PDPN. The first model included individual clinical diagnoses, the second included the CCI score, and the third the aDCSI. All models were adjusted for patient age group, sex, race, and region. Differences were statistically significant if the p-values were less than 0.05. All analyses were performed using the Instant Health Data platform (Panalgo, Boston, MA) and R version 3.2.1 (R Foundation for Statistical Computing, Vienna, Austria).

## Results

### Demographics and clinical characteristics

After applying the inclusion and exclusion criteria, 1,884,674 patients were included in the final analytic dataset (Fig. 2). Based on the prespecified definitions, 85.9% were categorized as no-DPN, 7.7% as DPN without pain, 2.4% as PDPN, and 4.0% as severe PDPN (Table 1). Mean ages ranged from 64.3 to 69.1 and the proportion of females in each cohort ranged from 46.6% to 61.6%. There was a higher proportion of Black patients in the severe PDPN cohort (21.3%) versus other cohorts (ranging from 14.9% to 16.1%, *p* < 0.0001).

**Table 1:** Baseline demographics & clinical characteristics

Measure	No-DPN	DPN	PDPN	Severe PDPN	*p*-value
N	1,619,323	146,107	44,432	74,812	
Age					<0.0001
Mean ± SD	64.3 ± 12.8	69.1 ± 10.5	67.9 ± 10.2	64.6 ± 10.4	
Median (IQR)	66 (57–73)	70 (65–77)	68 (62–75)	65 (58–72)	
Race					<0.0001
Asian	5.8%	4.4%	2.6%	1.4%	
Black	16.1%	14.9%	15.7%	21.3%	
White	61.3%	59.8%	63.7%	62.0%	
Hispanic	16.8%	20.8%	18.0%	15.3%	
Region					<0.0001
Midwest	19.7%	17.9%	19.7%	16.0%	
Northeast	14.2%	12.1%	10.5%	6.0%	
South	46.0%	45.8%	47.1%	58.9%	
West	20.2%	24.3%	22.7%	19.1%	
CCI score					<0.0001
Mean	0.9 ± 1.3	1.2 ± 1.4	1.4 ± 1.6	1.9 ± 1.8	
Median (IQR)	0 (0–1)	1 (0–2)	1 (0–2)	1 (0–3)	
aDCSI score					
Mean ± SD	1.1 ± 1.5	3 ± 1.9	3.2 ± 2	3.6 ± 3.6	<0.0001
Median (IQR)	0 (0–2)	3 (1–4)	3 (1–5)	3 (2–5)	
Diagnoses					
Diabetic foot ulcer	0.5%	4.9%	5.3%	8.5%	<0.0001
Loss of mobility^**^	8.1%	12.9%	19.1%	25.8%	<0.0001
Moderate to severe depression	1.1%	1.3%	2.3%	3.4%	<0.0001
Muscle weakness	5.8%	7.6%	12.3%	18.8%	<0.0001
Diabetic amyotrophy	0.0%	0.3%	0.3%	0.3%	<0.0001

*aDCSI* adapted diabetes complications severity index, *CCI* Charlson comorbidity index, *DPN* diabetic peripheral neuropathy, *PDPN* painful DPN

*Loss of mobility diagnoses includes: Abnormalities of gait and mobility, Ataxic gait, Paralytic gait, Difficulty in walking, not elsewhere classified, Other abnormalities of gait and mobility, Unsteadiness on feet, Other abnormalities of gait and mobility, Unspecified abnormalities of gait and mobility, Reduced mobility, confinement status and Other reduced mobility

Nearly all (93.6%) of study patients had diagnosis of type 2 diabetes, among whom 1,531,311 (86.8%) patients had no-DPN, 129,913 (7.4%) with DPN, 39,444 (2.2%) with PDPN and 63,991(3.6%) with severe PDPN (Appendix 1, Fig. S1). Fewer had diagnosis of type 1 diabetes (1.3%), among whom 22,513 (94.1%) had no-DPN, 1108 (4.6%) with DPN, 175 (0.7%) with PDPN and 141 (0.6%) with severe PDPN. The remaining 5.1% of patients with claims listing both diagnoses, therefore the exact diabetes type remained undetermined. When comparing presence of DPN by Type 1 and Type 2, patients with Type 2 diabetes had significantly more patients with PDPN and Severe DPN (*p* < 0.0001).

A higher proportion of patients with baseline diagnoses of anxiety, diabetic foot ulcers, loss of mobility, moderate to severe depression, and muscle weakness was observed in the PDPN and severe PDPN cohorts relative to no-DPN and DPN without pain (Table 1). Patients with severe PDPN were the most affected by these diagnoses (*p* < 0.0001). Mean CCI scores were comparatively higher among PDPN and severe PDPN patients (1.4 and 1.9, respectively), as were aDCSI scores (3.2 and 3.6, respectively).

### Resource use

Patients with severe PDPN had higher average number of all-cause medical visits during one year of follow-up than the other groups across all places of service (Table 2). The proportion of patients with severe PDPN with an ED visit was double that of patients with no-DPN alone (45.4% vs. 22.3%). Inpatient hospitalization for patients with severe PDPN was ~10% higher vs. patients with PDPN (26.4% vs 17.8%). The most common diagnoses (primary or secondary) listed on inpatient admissions were similar across cohorts, including hyperlipidemia, essential hypertension, atherosclerosis, and acute kidney failure. There were significantly more admissions with a pain-related diagnosis (primary or secondary) among those with severe PDPN (21.9%) versus patients with no DPN (9.0%).

**Table 2:** Medical resource use in follow-up year 1

All-cause medical resource use					
Measure	No-DPN	DPN	PDPN	Severe PDPN	*p*-value
N	1,619,323	146,107	44,432	74,812	
ED visits (%)	22.3%	24.5%	31.7%	45.4%	<0.0001
Conditional ED number visits					
Mean ± SD	1.7 ± 1.7	1.6 ± 1.3	1.8 ± 1.9	2.5 ± 2.9	<0.0001
Median (IQR)	1 (1–2)	1 (1–2)	1 (1–2)	1 (1–2)	
Inpatient hospital visits (%)	9.7%	13.4%	17.8%	26.4%	<0.0001
Conditional inpatient hospital number visits					
Mean ± SD	1.4 ± 0.8	1.4 ± 0.9	1.5 ± 1	1.7 ± 1.3	<0.0001
Median (IQR)	1 (1–1)	1 (1–1)	1 (1–1)	1 (1–1)	
Outpatient hospital visits (%)	53.3%	51.8%	62.4%	71.4%	<0.0001
Conditional outpatient hospital number visits					
Mean ± SD	4.8 ± 6.4	5.8 ± 8.1	7.2 ± 9.5	7.9 ± 9.9	<0.0001
Median (IQR)	3 (1–6)	3 (1–6)	3 (1–6)	3 (1–6)	
Office visits (%)	91.9%	92.6%	92.3%	94.4%	<0.0001
Conditional office number visits					
Mean ± SD	9.8 ± 9.3	11.9 ± 10.3	13.1 ± 11.4	16.3 ± 12.4	<0.0001
Median (IQR)	7 (4–13)	7 (4–13)	7 (4–13)	7 (4–13)	
Ambulatory surgical center visits (%)	9.9%	10.4%	11.4%	14.4%	<0.0001
Conditional ambulatory surgical center number visits					
Mean ± SD	1.5 ± 1.6	1.4 ± 1.6	1.5 ± 1.4	1.7 ± 1.5	<0.0001
Median (IQR)	1 (1–2)	1 (1–2)	1 (1–2)	1 (1–2)	
**Diabetes and diabetic neuropathy related medical-resource use**					
ED Visits (%)	12.8%	18.8%	25.2%	37.1%	<0.0001
Conditional ED number visits					
Mean ± SD	1.5 ± 1.3	1.5 ± 1.1	1.7 ± 1.7	2.2 ± 2.6	<0.0001
Median (IQR)	1 (1–2)	1 (1–2)	1 (1–2)	1 (1–2)	
Inpatient hospital visits (%)	7.9%	13.4%	17.7%	25.9%	<0.0001
Conditional inpatient hospital number visits					
Mean ± SD	1.3 ± 0.8	1.4 ± 0.9	1.5 ± 1	1.7 ± 1.3	<0.0001
Median (IQR)	1 (1–1)	1 (1–2)	1 (1–2)	1 (1–2)	
Outpatient hospital visits (%)	29.4%	36.4%	46.1%	53.0%	<0.0001
Conditional outpatient hospital number visits					
Mean ± SD	2.7 ± 3.1	3.7 ± 5.3	4.2 ± 6	4.5 ± 6.5	<0.0001
Median (IQR)	2 (1–3)	2 (1–4)	2 (1–5)	3 (1–5)	
Office visits (%)	69.0%	85.4%	84.3%	86.0%	<0.0001
Conditional office number visits					
Mean ± SD	4 ± 3.1	5.9 ± 4.7	6.1 ± 4.8	6.6 ± 5.2	<0.0001
Median (IQR)	3 (2–5)	5 (3–8)	5 (3–8)	5 (3–9)	
Ambulatory surgical center visits (%)	1.9%	3.0%	3.4%	4.2%	<0.0001
Conditional ambulatory surgical center number visits					
Mean ± SD	1.3 ± 0.7	1.3 ± 1.2	1.3 ± 0.9	1.3 ± 1.1	<0.0001
Median (IQR)	1(1–1)	1(1–1)	1(1–1)	1(1–1)	<0.0001
**Laboratory HbA1C values**					
HbA1C lab available (%)	44.9%	53.0%	51.2%	49.7%	<0.0001
Number of HbA1C labs					
Mean ± SD	2.0 ± 1.4	2.2 ± 1.5	2.2 ± 1.4	2.2 ± 1.8	<0.0001
Median (IQR)	2 (1–3)	2 (1–3)	2 (1–3)	2 (1–3)	
HbA1C results					
Mean ± SD	6.9 ± 2.9	7.3 ± 2.8	7.4 ± 3.5	7.5 ± 3.7	0.251
Median (IQR)	6.5 (5.9–7.4)	6.9 (6.2–7.9)	7.0 (6.2–8.0)	7.0 (6.2–8.2)	

*CGM* continuous glucose monitor, *DPN* diabetic peripheral neuropathy, *PDPN* painful DPN, *ED* emergency department

While nearly all patients had an office visit, the number of visits increased directly with increasing severity of disease, from a mean of 9.8 visits per patient per year for those with no-DPN to 16.3 per patient per year for those with severe PDPN (Table 2). While the magnitude of differences was lower when examining no-DPN or DPN without pain-related visits, trends were similar with increasing intensity of medical visits by severity of DPN.

Among patients with diagnosis of type 2 diabetes, the proportion with any prescription fill for insulin increased with advancing DPN severity, from 12.8% among those with no DPN to 43.5% among patients with severe PDPN (Table 2). The same trend was observed for presence of a prescription fill for a non-insulin anti-diabetic medication, from 53.6% among those with no DPN to 69.8% among severe PDPN. However, use of CGM among patients with type 1 diabetes decreased with advancing DPN severity (Appendix 1, Table S2).

In the subset of patients with lab data available (44.9%–55.0%) patients had on average 2.0–2.2 lab visits for a HbA1C test over year 1 of follow-up. The mean (SD) HbA1C result ranged from 6.9 (2.9) to 7.5 (3.7), while trending upward by DPN severity it was not statistically different across cohorts (*P* = 0.251).

### Payments

**Fig. 3:**
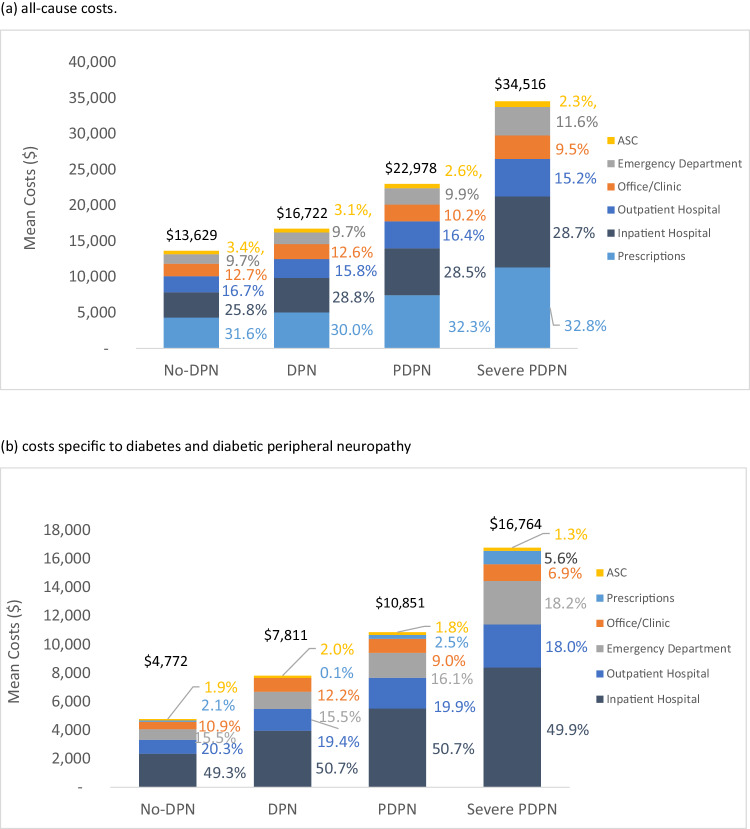
Mean costs at 1-year follow-up. **a** All-cause costs. **b** Costs specific to diabetes and diabetic peripheral neuropathy

**Fig. 4:**
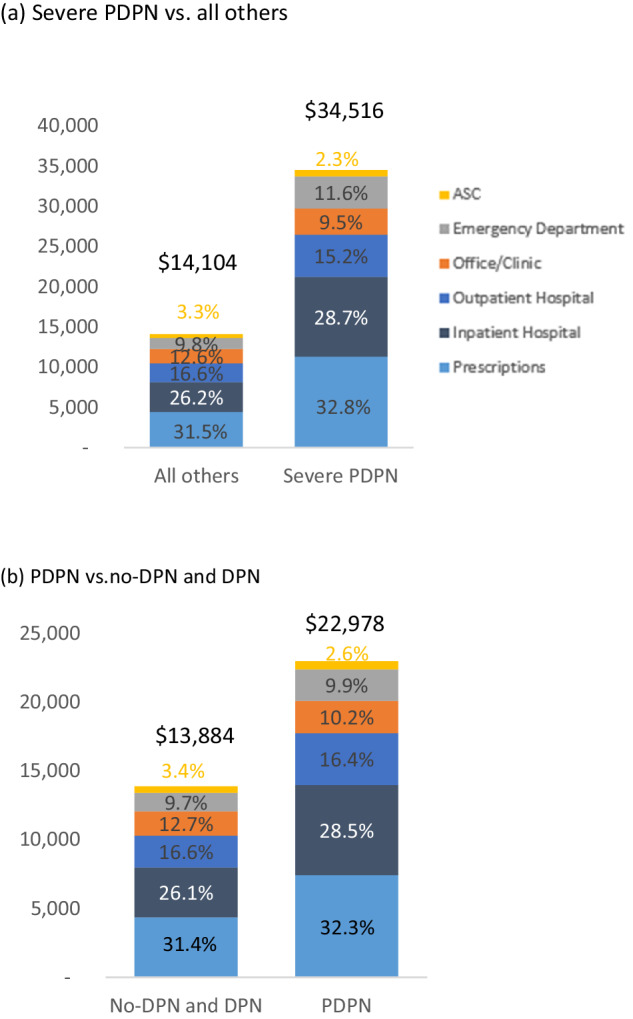
Sensitivity analyses: Mean 1 year follow-up costs, combined cohorts. **a** Severe PDPN vs. all others. **b** PDPN vs.no-DPN and DPN

The mean (SD) total all-cause cost associated with severe PDPN was $34,516 ($34,244), $22,978 ($27,979) for PDPN, $16,722 ($23,961) for DPN without pain and $13,629 ($21,504) for no-DPN (Fig. 3a, *p* < 0.0001). Prescription medication (30.0% to 32.8%) and inpatient costs (25.8% to 28.8%) made up the largest proportion of costs in all cohorts (Fig. 3a). Sensitivity analyses revealed that all-cause costs associated with severe PDPN were significantly higher compared to the costs for all other cohorts combined ($34,516 versus $14,104; *p* < 0.0001, Fig. 4a). The incremental cost of PDPN compared to combined no-DPN and DPN without pain was $9,094 (22,978 vs $13,884; *p* < 0.0001, Fig. 4b).

Mean (SD) total costs related to diabetes were $16,764 ($24,711) for severe PDPN, $10,851 ($20,334) for PDPN, $7,811 ($17,259) for DPN without pain, $4,772 ($13,323) for no-DPN (Fig. 3b, *p* < 0.0001). As with the all-cause analyses, the costs associated with severe PDPN were significantly higher relative to all other cohorts combined ($16,764 vs. $5,162, *p* < 0.0001).

### Multivariate regression models

Data were analyzed for factors associated with increased odds of severe PDPN using three models that evaluated either individual comorbidities or comorbidity burden based on CCI or aDSCI scores (Table 3). Factors associated with increased odds of severe PDPN, relative to no-DPN, DPN without pain, or PDPN included: age <75 years, Black race in models 1 and 2, anxiety, diabetic foot ulcer, loss of mobility, diabetic amyotrophy, moderate to severe depression, and muscle weakness diagnoses, as well as increases in CCI and aDCSI scores. Factors associated with decreased odds of severe PDPN included: Asian or Hispanic race, Black race in model 3, male sex, and living in the Midwest, Northeast, or West compared to the South.

**Table 3:** Factors associated with increased odds of classification as severe PDPN versus no-DPN, DPN without pain, or PDPN

Clinical characteristics	Model 1: Individual comorbidities		Model 2: CCI score		Model 3: aDCSI score	
	AOR	95% CI	AOR	95% CI	AOR	95% CI
Age (groups)						
18 to 54 vs 75+	1.44	1.40–1.48	1.75	1.70–1.80	3.39	3.29–3.48
55 to 64 vs 75+	2.41	2.36–2.47	2.63	2.57–2.69	3.86	3.77–3.96
65 to 74 vs 75+	1.44	1.41–1.48	1.51	1.47–1.54	1.83	1.79–1.87
Male	0.70	0.69–0.71	0.70	0.69–0.71	0.56	0.55–0.57
Race						
Asian	0.32	0.30–0.34	0.30	0.28–0.32	0.33	0.31–0.35
Black	1.12	1.09–1.14	1.06	1.04–1.08	0.97	0.95–0.99
Hispanic	0.89	0.87–0.91	0.83	0.81–0.85	0.77	0.75–0.79
Region						
Midwest vs. South	0.65	0.64–0.67	0.68	0.67–0.70	0.70	0.68–0.71
Northeast vs. South	0.34	0.33–0.35	0.38	0.37–0.39	0.40	0.38–0.41
West vs. South	0.88	0.86–0.90	0.85	0.83–0.87	0.85	0.83–0.86
Anxiety	1.93	1.88–1.98				
Diabetic amyotrophy	8.09	6.84–9.56				
Diabetic foot ulcer	6.54	6.32–6.76				
Loss of mobility	2.54	2.48–2.60				
Moderate to severe depression	1.87	1.78–1.96				
Muscle weakness	1.79	1.74–1.83				
Diabetes type						
Type 2 vs. 1	5.15	4.35–6.09				
Undetermined vs. 1	13.78	11.63–16.33				
CCI (zero reference case)						
1			2.60	2.55–2.66		
2			2.84	2.77–2.91		
3+			6.00	5.88–6.13		
aDSCI (0 and 1 are the reference)						
2					2.71	2.64–2.79
3					9.64	9.41–9.88
4					8.31	8.08–8.54
5					18.47	17.95–19.01
6+					30.25	29.45–31.08

*aDSCI* adapted diabetes complications severity index, *AOR* adjusted odds ratio, *CCI* Charlson comorbidity index

In model 1, diagnoses associated with the greatest odds of being classified in the severe PDPN cohort included diabetic amyotrophy and diabetic foot ulcer (Table 3). In model 2, a CCI score of 3 or higher was associated with a sixfold increase in the odds of being classified as severe PDPN relative to a CCI score of 0 (AOR = 6.00, 95% CI 5.88–6.13). In model 3, the of odds of being classified as severe PDPN increased exponentially as aDCSI score increased.

## Discussion

This research found that all-cause costs increased with worsening severity of diabetic neuropathy. Relative to patients without DPN, DPN without pain was associated with a $3,093 increase in all-cause, per-patient, per-year average annual costs. Costs associated with PDPN were $9,349 higher compared to no-DPN, and severe PDPN was associated with additional all-cause, annual per-patient costs of $20,887 relative to patients with diabetes alone. More than half of the costs for patients developing painful or severe PDPN were related to prescriptions and inpatient hospitalizations.

Prior retrospective research, using data collected in 2008–2013, has shown development of DPN is associated with one-year per-patient costs ranging from $12,492 for DPN without pain to $30,755 for severe PDPN [7]. Our present study findings confirmed similar all-cause costs of PDPN ($22,978) and severe PDPN ($34,516). The addition of our study is validation that these costs have remained consistent over time. Our findings also confirm that patients with a higher comorbidity burden are at higher probability of being classified as having severe PDPN [6, 9].

The relative sizes of each study cohort are notable: 85.9% with no-DPN, 7.7% with DPN without pain, 2.4% with PDPN, and 4.0% with severe PDPN. While it may seem counterintuitive that more patients were classified as having severe PDPN relative to PDPN, we speculate that if pain control treatments are effective, patients from this group are likely to move between states and there may be bias against remaining in the less-severe pain state due to the effectiveness of treatments. Our study design did not allow us to track the movement of patients between DPN states.

Compared to white patients, Black patients were at higher odds of being classified as severe PDPN in two of the three adjusted comorbidity models; this was not true for the other races evaluated. Black race is a known risk factor for diabetes; however current literature suggests that prevalence of PDPN symptoms is lower among Black patients [10]. Our differing outcomes could be due to differences in definitions or modeling methods, but further formal research is needed to further understand the extent and impact of racial disparities in diabetes and PDPN treatment.

For all cohorts, glucose control appeared to be well maintained from HbA1C lab results, however, this finding is likely not generalizable to the broader DPN population as our dataset only contained lab information for approximately half of patients. The correlation of HbA1c with severity of DPN is an area for future research. We observed a decrease in CGM utilization among patients with type 1 diabetes by DPN severity; however, it is unclear what is driving this correlation in absence of more detailed clinical notes data not available in claims. One hypothesis could be that use of CGM is associated with better diabetes control, and thus lower prevalence of complications such as DPN.

Controlling for patient demographics, regression analyses found that diagnosis of anxiety, moderate to severe depression, diabetic amyotrophy, diabetic foot ulcers, loss of mobility, and muscle weakness were significantly associated with higher odds of having severe PDPN. Similarly, patients with higher Charlson Comorbidity and aDCSI scores were significantly more likely to have severe PDPN. These findings support prior literature that greater comorbidity burden is associated with higher risk of diabetes complications [11].

Given our findings, it may be appropriate for physicians treating DPN to consider advanced treatment methods earlier in the treatment plan. Such treatments have been shown to help reduce pain and improve patient quality of life and may also reduce complications and costs [12–17]. At the same time, use of advanced nonpharmacologic treatments may help patients avoid the trial-and-error dosing of medications such as opioids, duloxetine, amitriptyline, and nortriptyline, and their associated, undesirable side effects.

The International Diabetes Federation guidelines for treatment of DPN provide an excellent framework for deciding how to manage PDPN [18], and physicians may consider accelerating the treatment lines if a particular patient isn’t responding to the current regimen.

## Limitations

This research has several limitations associated with the nature of retrospective claims data analyses, which can include miscoding and incorrect payments. Due to the retrospective nature of this analysis, causal relationships cannot be inferred, only associations between variables. These data are from a commercial claims database and are not representative of the entire United States diabetic population. This dataset did not contain details on patient socioeconomic status, which would be interesting in future research to explore relative to the prevalence of increasing severity of DPN. The claims data analyzed also resulted in some patients having evidence of both ICD-10 diagnoses of type 1 and 2 diabetes, despite these being alternative diagnoses to one another. Given the absence of specific clinical notes in claims data we had to categorize these patients as “Undetermined” diabetes type.

The main limitation of our study was the requirement that, due to a lack of specificity in diagnosis codes for neuropathy stratified for pain severity, as well as unavailability of pain score information in the database, we had to classify PDPN and severe PDPN using a composite algorithm of medication use and diagnosis codes. A literature review and expert opinion were used to identify diagnoses and medications that would feed into the algorithm.

Further, in the real world, these diagnoses are not static over time. Patients in the PDPN or severe PDPN groups who find effective treatment are likely to change states to DPN without severe pain, or without pain if effectively treated. Conversely, patients without adequate treatment may develop painful or severe painful DPN. An area for further research would be to design a model that can track individual patients’ progression between states to understand the effectiveness of specific treatments at controlling pain as well as associated costs. A related limitation was the possibility of capturing patients with other underlying causes of neuropathy, outside of diabetes. While we did exclude patients from analysis who had diagnosis of other causes of neuropathy (e.g., hereditary, drug or alcohol-induced, or due to toxic agents) this exclusionary list may not be comprehensive and is reliant on a patient having a diagnosis of other reason for neuropathy during the one-year baseline.

## Conclusion

This research shows that as diabetes and DPN complications increase, the cost burden to patients and US payers grows. Patients with painful DPN are more likely to experience debilitating conditions such as loss of mobility, diabetic foot ulcers and depression, and anxiety, which add to the cost burden and decrease patients’ quality of life. Our research also shows that current treatment options are costly to patients with advanced complications. Further research is needed to assess the effectiveness of various treatment options.

## Supplementary information

Supplemental Materials

## Abbreviations

aDCSI: adapted diabetes complications severity index
CCI: Charlson Comorbidity Index
CGM: continuous glucose monitor
DPN: diabetic peripheral neuropathy
PDPN: painful diabetic peripheral neuropathy
