# Supplementary material for: Healthcare costs and medical utilization patterns associated with painful and severe painful diabetic peripheral neuropathy
Source: Endocrine. 2024 Jul 13;86(3):1014–24. doi: 10.1007/s12020-024-03954-6 (PMC11554691; doi:10.1007/s12020-024-03954-6)
Supplement: Supplementary file 1 — Supplemental Materials [file 12020_2024_3954_MOESM1_ESM.docx]

Appendix 1: Supplemental Materials

Table S1.

| Diagnosis | Code Type | Code | Description |
| --- | --- | --- | --- |
| Anxiety | ICD-10 | F41.0 | Panic disorder [episodic paroxysmal anxiety] |
| Anxiety | ICD-10 | F41.1 | Generalized anxiety disorder |
| Depression | ICD-10 | F32.1 | Major depressive disorder, single episode, moderate |
| Depression | ICD-10 | F32.2 | Major depressive disorder, single episode, severe without psychotic features |
| Depression | ICD-10 | F32.3 | Major depressive disorder, single episode, severe with psychotic features |
| Diabetic Foot Ulcer | ICD-10 | E10.621 | Type 1 diabetes mellitus with foot ulcer |
| Diabetic Foot Ulcer | ICD-10 | E11.621 | Type 2 diabetes mellitus with foot ulcer |
| Diabetic Foot Ulcer | ICD-10 | E13.621 | Other specified diabetes mellitus with foot ulcer |
| Loss of Mobility | ICD-10 | R26 | R26 Abnormalities of gait and mobility [Non-Specific Code] |
| Loss of Mobility | ICD-10 | R26.0 | Ataxic gait |
| Loss of Mobility | ICD-10 | R26.1 | Paralytic gait |
| Loss of Mobility | ICD-10 | R26.2 | Difficulty in walking, not elsewhere classified |
| Loss of Mobility | ICD-10 | R26.8 | R26.8 Other abnormalities of gait and mobility [Non-Specific Code] |
| Loss of Mobility | ICD-10 | R26.81 | Unsteadiness on feet |
| Loss of Mobility | ICD-10 | R26.89 | Other abnormalities of gait and mobility |
| Loss of Mobility | ICD-10 | R26.9 | Unspecified abnormalities of gait and mobility |
| Loss of Mobility | ICD-10 | Z74.0 | Z74.0 Reduced mobility [Non-Specific Code] |
| Loss of Mobility | ICD-10 | Z74.01 | Bed confinement status |
| Loss of Mobility | ICD-10 | Z74.09 | Other reduced mobility |
| Muscle Weakness | ICD-10 | M62.81 | Muscle weakness (generalized) |
| Diabetic amyotrophy | ICD-10 | E10.44 | Type 1 diabetes mellitus with diabetic amyotrophy |
| Diabetic amyotrophy | ICD-10 | E11.44 | Type 2 diabetes mellitus with diabetic amyotrophy |
| Diabetic amyotrophy | ICD-10 | E13.44 | Other specified diabetes mellitus with diabetic amyotrophy |
| Diabetes Type 1 | ICD-10 | E10.10 | Type 1 diabetes mellitus with ketoacidosis without coma |
| Diabetes Type 1 | ICD-10 | E10.11 | Type 1 diabetes mellitus with ketoacidosis with coma |
| Diabetes Type 1 | ICD-10 | E10.21 | Type 1 diabetes mellitus with diabetic nephropathy |
| Diabetes Type 1 | ICD-10 | E10.22 | Type 1 diabetes mellitus with diabetic chronic kidney disease |
| Diabetes Type 1 | ICD-10 | E10.29 | Type 1 diabetes mellitus with other diabetic kidney complication |
| Diabetes Type 1 | ICD-10 | E10.311 | Type 1 diabetes mellitus with unspecified diabetic retinopathy with macular edema |
| Diabetes Type 1 | ICD-10 | E10.319 | Type 1 diabetes mellitus with unspecified diabetic retinopathy without macular edema |
| Diabetes Type 1 | ICD-10 | E10.3211 | Type 1 diabetes mellitus with mild nonproliferative diabetic retinopathy with macular edema, right eye |
| Diabetes Type 1 | ICD-10 | E10.3212 | Type 1 diabetes mellitus with mild nonproliferative diabetic retinopathy with macular edema, left eye |
| Diabetes Type 1 | ICD-10 | E10.3213 | Type 1 diabetes mellitus with mild nonproliferative diabetic retinopathy with macular edema, bilateral |
| Diabetes Type 1 | ICD-10 | E10.3219 | Type 1 diabetes mellitus with mild nonproliferative diabetic retinopathy with macular edema, unspecified eye |
| Diabetes Type 1 | ICD-10 | E10.3291 | Type 1 diabetes mellitus with mild nonproliferative diabetic retinopathy without macular edema, right eye |
| Diabetes Type 1 | ICD-10 | E10.3292 | Type 1 diabetes mellitus with mild nonproliferative diabetic retinopathy without macular edema, left eye |
| Diabetes Type 1 | ICD-10 | E10.3293 | Type 1 diabetes mellitus with mild nonproliferative diabetic retinopathy without macular edema, bilateral |
| Diabetes Type 1 | ICD-10 | E10.3299 | Type 1 diabetes mellitus with mild nonproliferative diabetic retinopathy without macular edema, unspecified eye |
| Diabetes Type 1 | ICD-10 | E10.3311 | Type 1 diabetes mellitus with moderate nonproliferative diabetic retinopathy with macular edema, right eye |
| Diabetes Type 1 | ICD-10 | E10.3312 | Type 1 diabetes mellitus with moderate nonproliferative diabetic retinopathy with macular edema, left eye |
| Diabetes Type 1 | ICD-10 | E10.3313 | Type 1 diabetes mellitus with moderate nonproliferative diabetic retinopathy with macular edema, bilateral |
| Diabetes Type 1 | ICD-10 | E10.3319 | Type 1 diabetes mellitus with moderate nonproliferative diabetic retinopathy with macular edema, unspecified eye |
| Diabetes Type 1 | ICD-10 | E10.3391 | Type 1 diabetes mellitus with moderate nonproliferative diabetic retinopathy without macular edema, right eye |
| Diabetes Type 1 | ICD-10 | E10.3392 | Type 1 diabetes mellitus with moderate nonproliferative diabetic retinopathy without macular edema, left eye |
| Diabetes Type 1 | ICD-10 | E10.3393 | Type 1 diabetes mellitus with moderate nonproliferative diabetic retinopathy without macular edema, bilateral |
| Diabetes Type 1 | ICD-10 | E10.3399 | Type 1 diabetes mellitus with moderate nonproliferative diabetic retinopathy without macular edema, unspecified eye |
| Diabetes Type 1 | ICD-10 | E10.3411 | Type 1 diabetes mellitus with severe nonproliferative diabetic retinopathy with macular edema, right eye |
| Diabetes Type 1 | ICD-10 | E10.3412 | Type 1 diabetes mellitus with severe nonproliferative diabetic retinopathy with macular edema, left eye |
| Diabetes Type 1 | ICD-10 | E10.3413 | Type 1 diabetes mellitus with severe nonproliferative diabetic retinopathy with macular edema, bilateral |
| Diabetes Type 1 | ICD-10 | E10.3419 | Type 1 diabetes mellitus with severe nonproliferative diabetic retinopathy with macular edema, unspecified eye |
| Diabetes Type 1 | ICD-10 | E10.3491 | Type 1 diabetes mellitus with severe nonproliferative diabetic retinopathy without macular edema, right eye |
| Diabetes Type 1 | ICD-10 | E10.3492 | Type 1 diabetes mellitus with severe nonproliferative diabetic retinopathy without macular edema, left eye |
| Diabetes Type 1 | ICD-10 | E10.3493 | Type 1 diabetes mellitus with severe nonproliferative diabetic retinopathy without macular edema, bilateral |
| Diabetes Type 1 | ICD-10 | E10.3499 | Type 1 diabetes mellitus with severe nonproliferative diabetic retinopathy without macular edema, unspecified eye |
| Diabetes Type 1 | ICD-10 | E10.3511 | Type 1 diabetes mellitus with proliferative diabetic retinopathy with macular edema, right eye |
| Diabetes Type 1 | ICD-10 | E10.3512 | Type 1 diabetes mellitus with proliferative diabetic retinopathy with macular edema, left eye |
| Diabetes Type 1 | ICD-10 | E10.3513 | Type 1 diabetes mellitus with proliferative diabetic retinopathy with macular edema, bilateral |
| Diabetes Type 1 | ICD-10 | E10.3519 | Type 1 diabetes mellitus with proliferative diabetic retinopathy with macular edema, unspecified eye |
| Diabetes Type 1 | ICD-10 | E10.3521 | Type 1 diabetes mellitus with proliferative diabetic retinopathy with traction retinal detachment involving the macula, right eye |
| Diabetes Type 1 | ICD-10 | E10.3522 | Type 1 diabetes mellitus with proliferative diabetic retinopathy with traction retinal detachment involving the macula, left eye |
| Diabetes Type 1 | ICD-10 | E10.3523 | Type 1 diabetes mellitus with proliferative diabetic retinopathy with traction retinal detachment involving the macula, bilateral |
| Diabetes Type 1 | ICD-10 | E10.3529 | Type 1 diabetes mellitus with proliferative diabetic retinopathy with traction retinal detachment involving the macula, unspecified eye |
| Diabetes Type 1 | ICD-10 | E10.3531 | Type 1 diabetes mellitus with proliferative diabetic retinopathy with traction retinal detachment not involving the macula, right eye |
| Diabetes Type 1 | ICD-10 | E10.3532 | Type 1 diabetes mellitus with proliferative diabetic retinopathy with traction retinal detachment not involving the macula, left eye |
| Diabetes Type 1 | ICD-10 | E10.3533 | Type 1 diabetes mellitus with proliferative diabetic retinopathy with traction retinal detachment not involving the macula, bilateral |
| Diabetes Type 1 | ICD-10 | E10.3539 | Type 1 diabetes mellitus with proliferative diabetic retinopathy with traction retinal detachment not involving the macula, unspecified eye |
| Diabetes Type 1 | ICD-10 | E10.3541 | Type 1 diabetes mellitus with proliferative diabetic retinopathy with combined traction retinal detachment and rhegmatogenous retinal detachment, right eye |
| Diabetes Type 1 | ICD-10 | E10.3542 | Type 1 diabetes mellitus with proliferative diabetic retinopathy with combined traction retinal detachment and rhegmatogenous retinal detachment, left eye |
| Diabetes Type 1 | ICD-10 | E10.3543 | Type 1 diabetes mellitus with proliferative diabetic retinopathy with combined traction retinal detachment and rhegmatogenous retinal detachment, bilateral |
| Diabetes Type 1 | ICD-10 | E10.3549 | Type 1 diabetes mellitus with proliferative diabetic retinopathy with combined traction retinal detachment and rhegmatogenous retinal detachment, unspecified eye |
| Diabetes Type 1 | ICD-10 | E10.3551 | Type 1 diabetes mellitus with stable proliferative diabetic retinopathy, right eye |
| Diabetes Type 1 | ICD-10 | E10.3552 | Type 1 diabetes mellitus with stable proliferative diabetic retinopathy, left eye |
| Diabetes Type 1 | ICD-10 | E10.3553 | Type 1 diabetes mellitus with stable proliferative diabetic retinopathy, bilateral |
| Diabetes Type 1 | ICD-10 | E10.3559 | Type 1 diabetes mellitus with stable proliferative diabetic retinopathy, unspecified eye |
| Diabetes Type 1 | ICD-10 | E10.3591 | Type 1 diabetes mellitus with proliferative diabetic retinopathy without macular edema, right eye |
| Diabetes Type 1 | ICD-10 | E10.3592 | Type 1 diabetes mellitus with proliferative diabetic retinopathy without macular edema, left eye |
| Diabetes Type 1 | ICD-10 | E10.3593 | Type 1 diabetes mellitus with proliferative diabetic retinopathy without macular edema, bilateral |
| Diabetes Type 1 | ICD-10 | E10.3599 | Type 1 diabetes mellitus with proliferative diabetic retinopathy without macular edema, unspecified eye |
| Diabetes Type 1 | ICD-10 | E10.36 | Type 1 diabetes mellitus with diabetic cataract |
| Diabetes Type 1 | ICD-10 | E10.37X1 | Type 1 diabetes mellitus with diabetic macular edema, resolved following treatment, right eye |
| Diabetes Type 1 | ICD-10 | E10.37X2 | Type 1 diabetes mellitus with diabetic macular edema, resolved following treatment, left eye |
| Diabetes Type 1 | ICD-10 | E10.37X3 | Type 1 diabetes mellitus with diabetic macular edema, resolved following treatment, bilateral |
| Diabetes Type 1 | ICD-10 | E10.37X9 | Type 1 diabetes mellitus with diabetic macular edema, resolved following treatment, unspecified eye |
| Diabetes Type 1 | ICD-10 | E10.39 | Type 1 diabetes mellitus with other diabetic ophthalmic complication |
| Diabetes Type 1 | ICD-10 | E10.40 | Type 1 diabetes mellitus with diabetic neuropathy, unspecified |
| Diabetes Type 1 | ICD-10 | E10.41 | Type 1 diabetes mellitus with diabetic mononeuropathy |
| Diabetes Type 1 | ICD-10 | E10.42 | Type 1 diabetes mellitus with diabetic polyneuropathy |
| Diabetes Type 1 | ICD-10 | E10.43 | Type 1 diabetes mellitus with diabetic autonomic (poly)neuropathy |
| Diabetes Type 1 | ICD-10 | E10.44 | Type 1 diabetes mellitus with diabetic amyotrophy |
| Diabetes Type 1 | ICD-10 | E10.49 | Type 1 diabetes mellitus with other diabetic neurological complication |
| Diabetes Type 1 | ICD-10 | E10.51 | Type 1 diabetes mellitus with diabetic peripheral angiopathy without gangrene |
| Diabetes Type 1 | ICD-10 | E10.52 | Type 1 diabetes mellitus with diabetic peripheral angiopathy with gangrene |
| Diabetes Type 1 | ICD-10 | E10.59 | Type 1 diabetes mellitus with other circulatory complications |
| Diabetes Type 1 | ICD-10 | E10.610 | Type 1 diabetes mellitus with diabetic neuropathic arthropathy |
| Diabetes Type 1 | ICD-10 | E10.618 | Type 1 diabetes mellitus with other diabetic arthropathy |
| Diabetes Type 1 | ICD-10 | E10.620 | Type 1 diabetes mellitus with diabetic dermatitis |
| Diabetes Type 1 | ICD-10 | E10.621 | Type 1 diabetes mellitus with foot ulcer |
| Diabetes Type 1 | ICD-10 | E10.622 | Type 1 diabetes mellitus with other skin ulcer |
| Diabetes Type 1 | ICD-10 | E10.628 | Type 1 diabetes mellitus with other skin complications |
| Diabetes Type 1 | ICD-10 | E10.630 | Type 1 diabetes mellitus with periodontal disease |
| Diabetes Type 1 | ICD-10 | E10.638 | Type 1 diabetes mellitus with other oral complications |
| Diabetes Type 1 | ICD-10 | E10.641 | Type 1 diabetes mellitus with hypoglycemia with coma |
| Diabetes Type 1 | ICD-10 | E10.649 | Type 1 diabetes mellitus with hypoglycemia without coma |
| Diabetes Type 1 | ICD-10 | E10.65 | Type 1 diabetes mellitus with hyperglycemia |
| Diabetes Type 1 | ICD-10 | E10.69 | Type 1 diabetes mellitus with other specified complication |
| Diabetes Type 1 | ICD-10 | E10.8 | Type 1 diabetes mellitus with unspecified complications |
| Diabetes Type 1 | ICD-10 | E10.9 | Type 1 diabetes mellitus without complications |
|  |  |  |  |
| Diabetes Type 2 | ICD-10 | E11.00 | Type 2 diabetes mellitus with hyperosmolarity without nonketotic hyperglycemic-hyperosmolar coma (NKHHC) |
| Diabetes Type 2 | ICD-10 | E11.01 | Type 2 diabetes mellitus with hyperosmolarity with coma |
| Diabetes Type 2 | ICD-10 | E11.10 | Type 2 diabetes mellitus with ketoacidosis without coma |
| Diabetes Type 2 | ICD-10 | E11.11 | Type 2 diabetes mellitus with ketoacidosis with coma |
| Diabetes Type 2 | ICD-10 | E11.21 | Type 2 diabetes mellitus with diabetic nephropathy |
| Diabetes Type 2 | ICD-10 | E11.22 | Type 2 diabetes mellitus with diabetic chronic kidney disease |
| Diabetes Type 2 | ICD-10 | E11.29 | Type 2 diabetes mellitus with other diabetic kidney complication |
| Diabetes Type 2 | ICD-10 | E11.311 | Type 2 diabetes mellitus with unspecified diabetic retinopathy with macular edema |
| Diabetes Type 2 | ICD-10 | E11.319 | Type 2 diabetes mellitus with unspecified diabetic retinopathy without macular edema |
| Diabetes Type 2 | ICD-10 | E11.3211 | Type 2 diabetes mellitus with mild nonproliferative diabetic retinopathy with macular edema, right eye |
| Diabetes Type 2 | ICD-10 | E11.3212 | Type 2 diabetes mellitus with mild nonproliferative diabetic retinopathy with macular edema, left eye |
| Diabetes Type 2 | ICD-10 | E11.3213 | Type 2 diabetes mellitus with mild nonproliferative diabetic retinopathy with macular edema, bilateral |
| Diabetes Type 2 | ICD-10 | E11.3219 | Type 2 diabetes mellitus with mild nonproliferative diabetic retinopathy with macular edema, unspecified eye |
| Diabetes Type 2 | ICD-10 | E11.3291 | Type 2 diabetes mellitus with mild nonproliferative diabetic retinopathy without macular edema, right eye |
| Diabetes Type 2 | ICD-10 | E11.3292 | Type 2 diabetes mellitus with mild nonproliferative diabetic retinopathy without macular edema, left eye |
| Diabetes Type 2 | ICD-10 | E11.3293 | Type 2 diabetes mellitus with mild nonproliferative diabetic retinopathy without macular edema, bilateral |
| Diabetes Type 2 | ICD-10 | E11.3299 | Type 2 diabetes mellitus with mild nonproliferative diabetic retinopathy without macular edema, unspecified eye |
| Diabetes Type 2 | ICD-10 | E11.3311 | Type 2 diabetes mellitus with moderate nonproliferative diabetic retinopathy with macular edema, right eye |
| Diabetes Type 2 | ICD-10 | E11.3312 | Type 2 diabetes mellitus with moderate nonproliferative diabetic retinopathy with macular edema, left eye |
| Diabetes Type 2 | ICD-10 | E11.3313 | Type 2 diabetes mellitus with moderate nonproliferative diabetic retinopathy with macular edema, bilateral |
| Diabetes Type 2 | ICD-10 | E11.3319 | Type 2 diabetes mellitus with moderate nonproliferative diabetic retinopathy with macular edema, unspecified eye |
| Diabetes Type 2 | ICD-10 | E11.3391 | Type 2 diabetes mellitus with moderate nonproliferative diabetic retinopathy without macular edema, right eye |
| Diabetes Type 2 | ICD-10 | E11.3392 | Type 2 diabetes mellitus with moderate nonproliferative diabetic retinopathy without macular edema, left eye |
| Diabetes Type 2 | ICD-10 | E11.3393 | Type 2 diabetes mellitus with moderate nonproliferative diabetic retinopathy without macular edema, bilateral |
| Diabetes Type 2 | ICD-10 | E11.3399 | Type 2 diabetes mellitus with moderate nonproliferative diabetic retinopathy without macular edema, unspecified eye |
| Diabetes Type 2 | ICD-10 | E11.3411 | Type 2 diabetes mellitus with severe nonproliferative diabetic retinopathy with macular edema, right eye |
| Diabetes Type 2 | ICD-10 | E11.3412 | Type 2 diabetes mellitus with severe nonproliferative diabetic retinopathy with macular edema, left eye |
| Diabetes Type 2 | ICD-10 | E11.3413 | Type 2 diabetes mellitus with severe nonproliferative diabetic retinopathy with macular edema, bilateral |
| Diabetes Type 2 | ICD-10 | E11.3419 | Type 2 diabetes mellitus with severe nonproliferative diabetic retinopathy with macular edema, unspecified eye |
| Diabetes Type 2 | ICD-10 | E11.3491 | Type 2 diabetes mellitus with severe nonproliferative diabetic retinopathy without macular edema, right eye |
| Diabetes Type 2 | ICD-10 | E11.3492 | Type 2 diabetes mellitus with severe nonproliferative diabetic retinopathy without macular edema, left eye |
| Diabetes Type 2 | ICD-10 | E11.3493 | Type 2 diabetes mellitus with severe nonproliferative diabetic retinopathy without macular edema, bilateral |
| Diabetes Type 2 | ICD-10 | E11.3499 | Type 2 diabetes mellitus with severe nonproliferative diabetic retinopathy without macular edema, unspecified eye |
| Diabetes Type 2 | ICD-10 | E11.3511 | Type 2 diabetes mellitus with proliferative diabetic retinopathy with macular edema, right eye |
| Diabetes Type 2 | ICD-10 | E11.3512 | Type 2 diabetes mellitus with proliferative diabetic retinopathy with macular edema, left eye |
| Diabetes Type 2 | ICD-10 | E11.3513 | Type 2 diabetes mellitus with proliferative diabetic retinopathy with macular edema, bilateral |
| Diabetes Type 2 | ICD-10 | E11.3519 | Type 2 diabetes mellitus with proliferative diabetic retinopathy with macular edema, unspecified eye |
| Diabetes Type 2 | ICD-10 | E11.3521 | Type 2 diabetes mellitus with proliferative diabetic retinopathy with traction retinal detachment involving the macula, right eye |
| Diabetes Type 2 | ICD-10 | E11.3522 | Type 2 diabetes mellitus with proliferative diabetic retinopathy with traction retinal detachment involving the macula, left eye |
| Diabetes Type 2 | ICD-10 | E11.3523 | Type 2 diabetes mellitus with proliferative diabetic retinopathy with traction retinal detachment involving the macula, bilateral |
| Diabetes Type 2 | ICD-10 | E11.3529 | Type 2 diabetes mellitus with proliferative diabetic retinopathy with traction retinal detachment involving the macula, unspecified eye |
| Diabetes Type 2 | ICD-10 | E11.3531 | Type 2 diabetes mellitus with proliferative diabetic retinopathy with traction retinal detachment not involving the macula, right eye |
| Diabetes Type 2 | ICD-10 | E11.3532 | Type 2 diabetes mellitus with proliferative diabetic retinopathy with traction retinal detachment not involving the macula, left eye |
| Diabetes Type 2 | ICD-10 | E11.3533 | Type 2 diabetes mellitus with proliferative diabetic retinopathy with traction retinal detachment not involving the macula, bilateral |
| Diabetes Type 2 | ICD-10 | E11.3539 | Type 2 diabetes mellitus with proliferative diabetic retinopathy with traction retinal detachment not involving the macula, unspecified eye |
| Diabetes Type 2 | ICD-10 | E11.3541 | Type 2 diabetes mellitus with proliferative diabetic retinopathy with combined traction retinal detachment and rhegmatogenous retinal detachment, right eye |
| Diabetes Type 2 | ICD-10 | E11.3542 | Type 2 diabetes mellitus with proliferative diabetic retinopathy with combined traction retinal detachment and rhegmatogenous retinal detachment, left eye |
| Diabetes Type 2 | ICD-10 | E11.3543 | Type 2 diabetes mellitus with proliferative diabetic retinopathy with combined traction retinal detachment and rhegmatogenous retinal detachment, bilateral |
| Diabetes Type 2 | ICD-10 | E11.3549 | Type 2 diabetes mellitus with proliferative diabetic retinopathy with combined traction retinal detachment and rhegmatogenous retinal detachment, unspecified eye |
| Diabetes Type 2 | ICD-10 | E11.3551 | Type 2 diabetes mellitus with stable proliferative diabetic retinopathy, right eye |
| Diabetes Type 2 | ICD-10 | E11.3552 | Type 2 diabetes mellitus with stable proliferative diabetic retinopathy, left eye |
| Diabetes Type 2 | ICD-10 | E11.3553 | Type 2 diabetes mellitus with stable proliferative diabetic retinopathy, bilateral |
| Diabetes Type 2 | ICD-10 | E11.3559 | Type 2 diabetes mellitus with stable proliferative diabetic retinopathy, unspecified eye |
| Diabetes Type 2 | ICD-10 | E11.3591 | Type 2 diabetes mellitus with proliferative diabetic retinopathy without macular edema, right eye |
| Diabetes Type 2 | ICD-10 | E11.3592 | Type 2 diabetes mellitus with proliferative diabetic retinopathy without macular edema, left eye |
| Diabetes Type 2 | ICD-10 | E11.3593 | Type 2 diabetes mellitus with proliferative diabetic retinopathy without macular edema, bilateral |
| Diabetes Type 2 | ICD-10 | E11.3599 | Type 2 diabetes mellitus with proliferative diabetic retinopathy without macular edema, unspecified eye |
| Diabetes Type 2 | ICD-10 | E11.36 | Type 2 diabetes mellitus with diabetic cataract |
| Diabetes Type 2 | ICD-10 | E11.37X1 | Type 2 diabetes mellitus with diabetic macular edema, resolved following treatment, right eye |
| Diabetes Type 2 | ICD-10 | E11.37X2 | Type 2 diabetes mellitus with diabetic macular edema, resolved following treatment, left eye |
| Diabetes Type 2 | ICD-10 | E11.37X3 | Type 2 diabetes mellitus with diabetic macular edema, resolved following treatment, bilateral |
| Diabetes Type 2 | ICD-10 | E11.37X9 | Type 2 diabetes mellitus with diabetic macular edema, resolved following treatment, unspecified eye |
| Diabetes Type 2 | ICD-10 | E11.39 | Type 2 diabetes mellitus with other diabetic ophthalmic complication |
| Diabetes Type 2 | ICD-10 | E11.40 | Type 2 diabetes mellitus with diabetic neuropathy, unspecified |
| Diabetes Type 2 | ICD-10 | E11.41 | Type 2 diabetes mellitus with diabetic mononeuropathy |
| Diabetes Type 2 | ICD-10 | E11.42 | Type 2 diabetes mellitus with diabetic polyneuropathy |
| Diabetes Type 2 | ICD-10 | E11.43 | Type 2 diabetes mellitus with diabetic autonomic (poly)neuropathy |
| Diabetes Type 2 | ICD-10 | E11.44 | Type 2 diabetes mellitus with diabetic amyotrophy |
| Diabetes Type 2 | ICD-10 | E11.49 | Type 2 diabetes mellitus with other diabetic neurological complication |
| Diabetes Type 2 | ICD-10 | E11.51 | Type 2 diabetes mellitus with diabetic peripheral angiopathy without gangrene |
| Diabetes Type 2 | ICD-10 | E11.52 | Type 2 diabetes mellitus with diabetic peripheral angiopathy with gangrene |
| Diabetes Type 2 | ICD-10 | E11.59 | Type 2 diabetes mellitus with other circulatory complications |
| Diabetes Type 2 | ICD-10 | E11.610 | Type 2 diabetes mellitus with diabetic neuropathic arthropathy |
| Diabetes Type 2 | ICD-10 | E11.618 | Type 2 diabetes mellitus with other diabetic arthropathy |
| Diabetes Type 2 | ICD-10 | E11.620 | Type 2 diabetes mellitus with diabetic dermatitis |
| Diabetes Type 2 | ICD-10 | E11.621 | Type 2 diabetes mellitus with foot ulcer |
| Diabetes Type 2 | ICD-10 | E11.622 | Type 2 diabetes mellitus with other skin ulcer |
| Diabetes Type 2 | ICD-10 | E11.628 | Type 2 diabetes mellitus with other skin complications |
| Diabetes Type 2 | ICD-10 | E11.630 | Type 2 diabetes mellitus with periodontal disease |
| Diabetes Type 2 | ICD-10 | E11.638 | Type 2 diabetes mellitus with other oral complications |
| Diabetes Type 2 | ICD-10 | E11.641 | Type 2 diabetes mellitus with hypoglycemia with coma |
| Diabetes Type 2 | ICD-10 | E11.649 | Type 2 diabetes mellitus with hypoglycemia without coma |
| Diabetes Type 2 | ICD-10 | E11.65 | Type 2 diabetes mellitus with hyperglycemia |
| Diabetes Type 2 | ICD-10 | E11.69 | Type 2 diabetes mellitus with other specified complication |
| Diabetes Type 2 | ICD-10 | E11.8 | Type 2 diabetes mellitus with unspecified complications |
| Diabetes Type 2 | ICD-10 | E11.9 | Type 2 diabetes mellitus without complications |
| Diabetes Type 2 | ICD-10 | E13.10 | Other specified diabetes mellitus with ketoacidosis without coma |
| Diabetic Neuropathy | ICD-10 | E10.43 | Type 1 diabetes mellitus with diabetic autonomic (poly)neuropathy |
| Diabetic Neuropathy | ICD-11 | E11.43 | Type 2 diabetes mellitus with diabetic autonomic (poly)neuropathy |
| Diabetic Neuropathy | ICD-12 | E10.44 | Type 1 diabetes mellitus with diabetic amyotrophy |
| Diabetic Neuropathy | ICD-13 | E11.44 | Type 2 diabetes mellitus with diabetic amyotrophy |
| Diabetic Neuropathy | ICD-14 | E08.41 | Diabetes mellitus due to underlying condition with diabetic mononeuropathy |
| Diabetic Neuropathy | ICD-15 | E09.41 | Drug or chemical induced diabetes mellitus with neurological complications with diabetic mononeuropathy |
| Diabetic Neuropathy | ICD-16 | E10.41 | Type 1 diabetes mellitus with diabetic mononeuropathy |
| Diabetic Neuropathy | ICD-17 | E11.41 | Type 2 diabetes mellitus with diabetic mononeuropathy |
| Diabetic Neuropathy | ICD-18 | E13.41 | Other specified diabetes mellitus with diabetic mononeuropathy |
| Diabetic Neuropathy | ICD-19 | E08.42 | Diabetes mellitus due to underlying condition with diabetic polyneuropathy |
| Diabetic Neuropathy | ICD-20 | E09.42 | Drug or chemical induced diabetes mellitus with neurological complications with diabetic polyneuropathy |
| Diabetic Neuropathy | ICD-21 | E10.42 | Type 1 diabetes mellitus with diabetic polyneuropathy |
| Diabetic Neuropathy | ICD-22 | E11.42 | Type 2 diabetes mellitus with diabetic polyneuropathy |
| Diabetic Neuropathy | ICD-23 | E13.42 | Other specified diabetes mellitus with diabetic polyneuropathy |
| Diabetic Neuropathy | ICD-24 | E10.40 | Type 1 diabetes mellitus with diabetic neuropathy, unspecified |
| Diabetic Neuropathy | ICD-25 | E11.40 | Type 2 diabetes mellitus with diabetic neuropathy, unspecified |
|  |  |  |  |
| Spinal cord stimulation procedure | CPT | 63661 | Removal of spinal neurostimulator electrode percutaneous array(s), including fluoroscopy, when performed |
| Spinal cord stimulation procedure | CPT | 63662 | Removal of spinal neurostimulator electrode plate/paddle(s) placed via laminotomy or laminectomy, including fluoroscopy, when performed |
| Spinal cord stimulation procedure | ICD-10 | 00PU0MZ | Removal of Neurostimulator Lead from Spinal Canal, Open Approach |
| Spinal cord stimulation procedure | ICD-10 | 00PU3MZ | Removal of Neurostimulator Lead from Spinal Canal, Percutaneous Approach |
| Spinal cord stimulation procedure | ICD-10 | 00PV0MZ | Removal of Neurostimulator Lead from Spinal Cord, Open Approach |
| Spinal cord stimulation procedure | ICD-10 | 00PV3MZ | Removal of Neurostimulator Lead from Spinal Cord, Percutaneous Approach |
| Spinal cord stimulation procedure | CPT | 63663 | Revision including replacement, when performed, of spinal neurostimulator electrode percutaneous array(s), including fluoroscopy, when performed |
| Spinal cord stimulation procedure | CPT | 63664 | Revision including replacement, when performed, of spinal neurostimulator electrode plate/paddle(s) placed via laminotomy or laminectomy, including fluoroscopy, when performed |
| Spinal cord stimulation procedure | ICD-10 | 00WU0MZ | Revision of Neurostimulator Lead in Spinal Canal, Open Approach |
| Spinal cord stimulation procedure | ICD-10 | 00WU3MZ | Revision of Neurostimulator Lead in Spinal Canal, Percutaneous Approach |
| Spinal cord stimulation procedure | ICD-10 | 00WV0MZ | Revision of Neurostimulator Lead in Spinal Cord, Open Approach |
| Spinal cord stimulation procedure | ICD-10 | 00WV3MZ | Revision of Neurostimulator Lead in Spinal Cord, Percutaneous Approach |
| Spinal cord stimulation procedure | ICD-10 | 00HU0MZ | Insertion of Neurostimulator Lead into Spinal Canal, Open Approach |
| Spinal cord stimulation procedure | ICD-10 | 00HU3MZ | Insertion of Neurostimulator Lead into Spinal Canal, Percutaneous Approach |
| Spinal cord stimulation procedure | ICD-10 | 00HV0MZ | Insertion of Neurostimulator Lead into Spinal Cord, Open Approach |
| Spinal cord stimulation procedure | ICD-10 | 00HV3MZ | Insertion of Neurostimulator Lead into Spinal Cord, Percutaneous Approach |
| Spinal cord stimulation procedure | CPT | 63650 | Percutaneous implantation of neurostimulator electrode array, epidural |
| Spinal cord stimulation procedure | CPT | 63685 | Insertion or replacement of spinal neurostimulator pulse generator or receiver, direct or inductive coupling |
| Spinal cord stimulation procedure | ICD-10 | 0JH70BZ | Insertion of Single Array Stimulator Generator into Back Subcutaneous Tissue and Fascia, Open Approach |
| Spinal cord stimulation procedure | ICD-10 | 0JH70CZ | Insertion of Single Array Rechargeable Stimulator Generator into Back Subcutaneous Tissue and Fascia, Open Approach |
| Spinal cord stimulation procedure | ICD-10 | 0JH70DZ | Insertion of Multiple Array Stimulator Generator into Back Subcutaneous Tissue and Fascia, Open Approach |
| Spinal cord stimulation procedure | ICD-10 | 0JH70EZ | Insertion of Multiple Array Rechargeable Stimulator Generator into Back Subcutaneous Tissue and Fascia, Open Approach |
| Spinal cord stimulation procedure | ICD-10 | 0JH80BZ | Insertion of Single Array Stimulator Generator into Abdomen Subcutaneous Tissue and Fascia, Open Approach |
| Spinal cord stimulation procedure | ICD-10 | 0JH80CZ | Insertion of Single Array Rechargeable Stimulator Generator into Abdomen Subcutaneous Tissue and Fascia, Open Approach |
| Spinal cord stimulation procedure | ICD-10 | 0JH80DZ | Insertion of Multiple Array Stimulator Generator into Abdomen Subcutaneous Tissue and Fascia, Open Approach |
| Spinal cord stimulation procedure | ICD-10 | 0JH80EZ | Insertion of Multiple Array Rechargeable Stimulator Generator into Abdomen Subcutaneous Tissue and Fascia, Open Approach |
| Spinal cord stimulation procedure | ICD-10 | 0JPT0MZ | Removal of Stimulator Generator from Trunk Subcutaneous Tissue and Fascia, Open Approach |
| Spinal cord stimulation procedure | ICD-10 | 0JPT3MZ | Removal of Stimulator Generator from Trunk Subcutaneous Tissue and Fascia, Percutaneous Approach |
| Spinal cord stimulation procedure | ICD-10 | 0JWT0MZ | Revision of Stimulator Generator in Trunk Subcutaneous Tissue and Fascia, Open Approach |
| Spinal cord stimulation procedure | ICD-10 | 0JWT3MZ | Revision of Stimulator Generator in Trunk Subcutaneous Tissue and Fascia, Percutaneous Approach |
| Spinal cord stimulation procedure | ICD-10 | 0JWTXMZ | Revision of Stimulator Generator in Trunk Subcutaneous Tissue and Fascia, External Approach |
| Spinal cord stimulation procedure | CPT | 63688 | Revision or removal of implanted spinal neurostimulator pulse generator or receiver |
| Spinal cord stimulation procedure | CPT | 63655 | Laminectomy for implantation of neurostimulator electrodes, plate/paddle, epidural |
| Targetted drug delivery procedure | CPT | 62369 | Electronic analysis of programmable, implanted pump for intrathecal or epidural drug infusion (includes evaluation of reservoir status, alarm status, drug prescription status); with reprogramming and refill |
| Targetted drug delivery procedure | CPT | 62370 | Electronic analysis of programmable, implanted pump for intrathecal or epidural drug infusion (includes evaluation of reservoir status, alarm status, drug prescription status); with reprogramming and refill (requiring skill of a physician or other qualified health care professional) |
| Targetted drug delivery procedure | CPT | 95990 | Refilling and maintenance of implantable pump or reservoir for drug delivery, spinal (intrathecal, epidural) or brain (intraventricular), includes electronic analysis of pump, when performed; |
| Targetted drug delivery procedure | CPT | 95991 | Refilling and maintenance of implantable pump or reservoir for drug delivery, spinal (intrathecal, epidural) or brain (intraventricular), includes electronic analysis of pump, when performed; requiring skill of a physician or other qualified health care professional |
| Targetted drug delivery procedure | CPT | 62362 | Implantation or replacement of device for intrathecal or epidural drug infusion; programmable pump, including preparation of pump, with or without programming |
| Targetted drug delivery procedure | ICD-10 | 0JH80VZ | Insertion of Infusion Pump into Abdomen Subcutaneous Tissue and Fascia, Open Approach |
| Targetted drug delivery procedure | CPT | S9328 | Home infusion therapy, implanted pump pain management infusion; administrative services, professional pharmacy services, care coordination, and all necessary supplies and equipment (drugs and nursing visits coded separately), per diem |
| Targetted drug delivery procedure | CPT | 62365 | Removal of subcutaneous reservoir or pump, previously implanted for intrathecal or epidural infusion |
| Targetted drug delivery procedure | ICD-10 | 00PU03Z | Removal of Infusion Device from Spinal Canal, Open Approach |
| Targetted drug delivery procedure | ICD-10 | 00PU33Z | Removal of Infusion Device from Spinal Canal, Percutaneous Approach |
| Targetted drug delivery procedure | ICD-10 | 00PU43Z | Removal of Infusion Device from Spinal Canal, Percutaneous Endoscopic Approach |
| Targetted drug delivery procedure | ICD-10 | 00PUX3Z | Removal of Infusion Device from Spinal Canal, External Approach |
| Targetted drug delivery procedure | ICD-10 | 0JPT0VZ | Removal of Infusion Pump from Trunk Subcutaneous Tissue and Fascia, Open Approach |
| Targetted drug delivery procedure | ICD-10 | 0JPT3VZ | Removal of Infusion Pump from Trunk Subcutaneous Tissue and Fascia, Percutaneous Approach |
| Targetted drug delivery procedure | CPT | 62350 | Implantation, revision or repositioning of tunneled intrathecal or epidural catheter, for long-term medication administration via an external pump or implantable reservoir/infusion pump; without laminectomy |
| Targetted drug delivery procedure | CPT | 62351 | Implantation, revision or repositioning of tunneled intrathecal or epidural catheter, for long-term medication administration via an external pump or implantable reservoir/infusion pump; with laminectomy |
| Targetted drug delivery procedure | ICD-10 | 00HU33Z | Insertion of Infusion Device into Spinal Canal, Percutaneous Approach |
| Cancer Diagnosis | ICD-10 | C00.0 | Malignant neoplasm of external upper lip |
| Cancer Diagnosis | ICD-10 | C00.1 | Malignant neoplasm of external lower lip |
| Cancer Diagnosis | ICD-10 | C00.2 | Malignant neoplasm of external lip, unspecified |
| Cancer Diagnosis | ICD-10 | C00.3 | Malignant neoplasm of upper lip, inner aspect |
| Cancer Diagnosis | ICD-10 | C00.4 | Malignant neoplasm of lower lip, inner aspect |
| Cancer Diagnosis | ICD-10 | C00.5 | Malignant neoplasm of lip, unspecified, inner aspect |
| Cancer Diagnosis | ICD-10 | C00.6 | Malignant neoplasm of commissure of lip, unspecified |
| Cancer Diagnosis | ICD-10 | C00.8 | Malignant neoplasm of overlapping sites of lip |
| Cancer Diagnosis | ICD-10 | C00.9 | Malignant neoplasm of lip, unspecified |
| Cancer Diagnosis | ICD-10 | C01 | Malignant neoplasm of base of tongue |
| Cancer Diagnosis | ICD-10 | C02.0 | Malignant neoplasm of dorsal surface of tongue |
| Cancer Diagnosis | ICD-10 | C02.1 | Malignant neoplasm of border of tongue |
| Cancer Diagnosis | ICD-10 | C02.2 | Malignant neoplasm of ventral surface of tongue |
| Cancer Diagnosis | ICD-10 | C02.3 | Malignant neoplasm of anterior two-thirds of tongue, part unspecified |
| Cancer Diagnosis | ICD-10 | C02.4 | Malignant neoplasm of lingual tonsil |
| Cancer Diagnosis | ICD-10 | C02.8 | Malignant neoplasm of overlapping sites of tongue |
| Cancer Diagnosis | ICD-10 | C02.9 | Malignant neoplasm of tongue, unspecified |
| Cancer Diagnosis | ICD-10 | C03.0 | Malignant neoplasm of upper gum |
| Cancer Diagnosis | ICD-10 | C03.1 | Malignant neoplasm of lower gum |
| Cancer Diagnosis | ICD-10 | C03.9 | Malignant neoplasm of gum, unspecified |
| Cancer Diagnosis | ICD-10 | C04.0 | Malignant neoplasm of anterior floor of mouth |
| Cancer Diagnosis | ICD-10 | C04.1 | Malignant neoplasm of lateral floor of mouth |
| Cancer Diagnosis | ICD-10 | C04.8 | Malignant neoplasm of overlapping sites of floor of mouth |
| Cancer Diagnosis | ICD-10 | C04.9 | Malignant neoplasm of floor of mouth, unspecified |
| Cancer Diagnosis | ICD-10 | C05.0 | Malignant neoplasm of hard palate |
| Cancer Diagnosis | ICD-10 | C05.1 | Malignant neoplasm of soft palate |
| Cancer Diagnosis | ICD-10 | C05.2 | Malignant neoplasm of uvula |
| Cancer Diagnosis | ICD-10 | C05.8 | Malignant neoplasm of overlapping sites of palate |
| Cancer Diagnosis | ICD-10 | C05.9 | Malignant neoplasm of palate, unspecified |
| Cancer Diagnosis | ICD-10 | C06.0 | Malignant neoplasm of cheek mucosa |
| Cancer Diagnosis | ICD-10 | C06.1 | Malignant neoplasm of vestibule of mouth |
| Cancer Diagnosis | ICD-10 | C06.2 | Malignant neoplasm of retromolar area |
| Cancer Diagnosis | ICD-10 | C06.80 | Malignant neoplasm of overlapping sites of unspecified parts of mouth |
| Cancer Diagnosis | ICD-10 | C06.89 | Malignant neoplasm of overlapping sites of other parts of mouth |
| Cancer Diagnosis | ICD-10 | C06.9 | Malignant neoplasm of mouth, unspecified |
| Cancer Diagnosis | ICD-10 | C07 | Malignant neoplasm of parotid gland |
| Cancer Diagnosis | ICD-10 | C08.0 | Malignant neoplasm of submandibular gland |
| Cancer Diagnosis | ICD-10 | C08.1 | Malignant neoplasm of sublingual gland |
| Cancer Diagnosis | ICD-10 | C08.9 | Malignant neoplasm of major salivary gland, unspecified |
| Cancer Diagnosis | ICD-10 | C09.0 | Malignant neoplasm of tonsillar fossa |
| Cancer Diagnosis | ICD-10 | C09.1 | Malignant neoplasm of tonsillar pillar (anterior) (posterior) |
| Cancer Diagnosis | ICD-10 | C09.8 | Malignant neoplasm of overlapping sites of tonsil |
| Cancer Diagnosis | ICD-10 | C09.9 | Malignant neoplasm of tonsil, unspecified |
| Cancer Diagnosis | ICD-10 | C10.0 | Malignant neoplasm of vallecula |
| Cancer Diagnosis | ICD-10 | C10.1 | Malignant neoplasm of anterior surface of epiglottis |
| Cancer Diagnosis | ICD-10 | C10.2 | Malignant neoplasm of lateral wall of oropharynx |
| Cancer Diagnosis | ICD-10 | C10.3 | Malignant neoplasm of posterior wall of oropharynx |
| Cancer Diagnosis | ICD-10 | C10.4 | Malignant neoplasm of branchial cleft |
| Cancer Diagnosis | ICD-10 | C10.8 | Malignant neoplasm of overlapping sites of oropharynx |
| Cancer Diagnosis | ICD-10 | C10.9 | Malignant neoplasm of oropharynx, unspecified |
| Cancer Diagnosis | ICD-10 | C11.0 | Malignant neoplasm of superior wall of nasopharynx |
| Cancer Diagnosis | ICD-10 | C11.1 | Malignant neoplasm of posterior wall of nasopharynx |
| Cancer Diagnosis | ICD-10 | C11.2 | Malignant neoplasm of lateral wall of nasopharynx |
| Cancer Diagnosis | ICD-10 | C11.3 | Malignant neoplasm of anterior wall of nasopharynx |
| Cancer Diagnosis | ICD-10 | C11.8 | Malignant neoplasm of overlapping sites of nasopharynx |
| Cancer Diagnosis | ICD-10 | C11.9 | Malignant neoplasm of nasopharynx, unspecified |
| Cancer Diagnosis | ICD-10 | C12 | Malignant neoplasm of pyriform sinus |
| Cancer Diagnosis | ICD-10 | C13.0 | Malignant neoplasm of postcricoid region |
| Cancer Diagnosis | ICD-10 | C13.1 | Malignant neoplasm of aryepiglottic fold, hypopharyngeal aspect |
| Cancer Diagnosis | ICD-10 | C13.2 | Malignant neoplasm of posterior wall of hypopharynx |
| Cancer Diagnosis | ICD-10 | C13.8 | Malignant neoplasm of overlapping sites of hypopharynx |
| Cancer Diagnosis | ICD-10 | C13.9 | Malignant neoplasm of hypopharynx, unspecified |
| Cancer Diagnosis | ICD-10 | C14.0 | Malignant neoplasm of pharynx, unspecified |
| Cancer Diagnosis | ICD-10 | C14.2 | Malignant neoplasm of Waldeyer's ring |
| Cancer Diagnosis | ICD-10 | C14.8 | Malignant neoplasm of overlapping sites of lip, oral cavity and pharynx |
| Cancer Diagnosis | ICD-10 | C15.3 | Malignant neoplasm of upper third of esophagus |
| Cancer Diagnosis | ICD-10 | C15.4 | Malignant neoplasm of middle third of esophagus |
| Cancer Diagnosis | ICD-10 | C15.5 | Malignant neoplasm of lower third of esophagus |
| Cancer Diagnosis | ICD-10 | C15.8 | Malignant neoplasm of overlapping sites of esophagus |
| Cancer Diagnosis | ICD-10 | C15.9 | Malignant neoplasm of esophagus, unspecified |
| Cancer Diagnosis | ICD-10 | C16.0 | Malignant neoplasm of cardia |
| Cancer Diagnosis | ICD-10 | C16.1 | Malignant neoplasm of fundus of stomach |
| Cancer Diagnosis | ICD-10 | C16.2 | Malignant neoplasm of body of stomach |
| Cancer Diagnosis | ICD-10 | C16.3 | Malignant neoplasm of pyloric antrum |
| Cancer Diagnosis | ICD-10 | C16.4 | Malignant neoplasm of pylorus |
| Cancer Diagnosis | ICD-10 | C16.5 | Malignant neoplasm of lesser curvature of stomach, unspecified |
| Cancer Diagnosis | ICD-10 | C16.6 | Malignant neoplasm of greater curvature of stomach, unspecified |
| Cancer Diagnosis | ICD-10 | C16.8 | Malignant neoplasm of overlapping sites of stomach |
| Cancer Diagnosis | ICD-10 | C16.9 | Malignant neoplasm of stomach, unspecified |
| Cancer Diagnosis | ICD-10 | C17.0 | Malignant neoplasm of duodenum |
| Cancer Diagnosis | ICD-10 | C17.1 | Malignant neoplasm of jejunum |
| Cancer Diagnosis | ICD-10 | C17.2 | Malignant neoplasm of ileum |
| Cancer Diagnosis | ICD-10 | C17.3 | Meckel's diverticulum, malignant |
| Cancer Diagnosis | ICD-10 | C17.8 | Malignant neoplasm of overlapping sites of small intestine |
| Cancer Diagnosis | ICD-10 | C17.9 | Malignant neoplasm of small intestine, unspecified |
| Cancer Diagnosis | ICD-10 | C18.0 | Malignant neoplasm of cecum |
| Cancer Diagnosis | ICD-10 | C18.1 | Malignant neoplasm of appendix |
| Cancer Diagnosis | ICD-10 | C18.2 | Malignant neoplasm of ascending colon |
| Cancer Diagnosis | ICD-10 | C18.3 | Malignant neoplasm of hepatic flexure |
| Cancer Diagnosis | ICD-10 | C18.4 | Malignant neoplasm of transverse colon |
| Cancer Diagnosis | ICD-10 | C18.5 | Malignant neoplasm of splenic flexure |
| Cancer Diagnosis | ICD-10 | C18.6 | Malignant neoplasm of descending colon |
| Cancer Diagnosis | ICD-10 | C18.7 | Malignant neoplasm of sigmoid colon |
| Cancer Diagnosis | ICD-10 | C18.8 | Malignant neoplasm of overlapping sites of colon |
| Cancer Diagnosis | ICD-10 | C18.9 | Malignant neoplasm of colon, unspecified |
| Cancer Diagnosis | ICD-10 | C19 | Malignant neoplasm of rectosigmoid junction |
| Cancer Diagnosis | ICD-10 | C20 | Malignant neoplasm of rectum |
| Cancer Diagnosis | ICD-10 | C21.0 | Malignant neoplasm of anus, unspecified |
| Cancer Diagnosis | ICD-10 | C21.1 | Malignant neoplasm of anal canal |
| Cancer Diagnosis | ICD-10 | C21.2 | Malignant neoplasm of cloacogenic zone |
| Cancer Diagnosis | ICD-10 | C21.8 | Malignant neoplasm of overlapping sites of rectum, anus and anal canal |
| Cancer Diagnosis | ICD-10 | C22.0 | Liver cell carcinoma |
| Cancer Diagnosis | ICD-10 | C22.1 | Intrahepatic bile duct carcinoma |
| Cancer Diagnosis | ICD-10 | C22.2 | Hepatoblastoma |
| Cancer Diagnosis | ICD-10 | C22.3 | Angiosarcoma of liver |
| Cancer Diagnosis | ICD-10 | C22.4 | Other sarcomas of liver |
| Cancer Diagnosis | ICD-10 | C22.7 | Other specified carcinomas of liver |
| Cancer Diagnosis | ICD-10 | C22.8 | Malignant neoplasm of liver, primary, unspecified as to type |
| Cancer Diagnosis | ICD-10 | C22.9 | Malignant neoplasm of liver, not specified as primary or secondary |
| Cancer Diagnosis | ICD-10 | C23 | Malignant neoplasm of gallbladder |
| Cancer Diagnosis | ICD-10 | C24.0 | Malignant neoplasm of extrahepatic bile duct |
| Cancer Diagnosis | ICD-10 | C24.1 | Malignant neoplasm of ampulla of Vater |
| Cancer Diagnosis | ICD-10 | C24.8 | Malignant neoplasm of overlapping sites of biliary tract |
| Cancer Diagnosis | ICD-10 | C24.9 | Malignant neoplasm of biliary tract, unspecified |
| Cancer Diagnosis | ICD-10 | C25.0 | Malignant neoplasm of head of pancreas |
| Cancer Diagnosis | ICD-10 | C25.1 | Malignant neoplasm of body of pancreas |
| Cancer Diagnosis | ICD-10 | C25.2 | Malignant neoplasm of tail of pancreas |
| Cancer Diagnosis | ICD-10 | C25.3 | Malignant neoplasm of pancreatic duct |
| Cancer Diagnosis | ICD-10 | C25.4 | Malignant neoplasm of endocrine pancreas |
| Cancer Diagnosis | ICD-10 | C25.7 | Malignant neoplasm of other parts of pancreas |
| Cancer Diagnosis | ICD-10 | C25.8 | Malignant neoplasm of overlapping sites of pancreas |
| Cancer Diagnosis | ICD-10 | C25.9 | Malignant neoplasm of pancreas, unspecified |
| Cancer Diagnosis | ICD-10 | C26.0 | Malignant neoplasm of intestinal tract, part unspecified |
| Cancer Diagnosis | ICD-10 | C26.1 | Malignant neoplasm of spleen |
| Cancer Diagnosis | ICD-10 | C26.9 | Malignant neoplasm of ill-defined sites within the digestive system |
| Cancer Diagnosis | ICD-10 | C30.0 | Malignant neoplasm of nasal cavity |
| Cancer Diagnosis | ICD-10 | C30.1 | Malignant neoplasm of middle ear |
| Cancer Diagnosis | ICD-10 | C31.0 | Malignant neoplasm of maxillary sinus |
| Cancer Diagnosis | ICD-10 | C31.1 | Malignant neoplasm of ethmoidal sinus |
| Cancer Diagnosis | ICD-10 | C31.2 | Malignant neoplasm of frontal sinus |
| Cancer Diagnosis | ICD-10 | C31.3 | Malignant neoplasm of sphenoid sinus |
| Cancer Diagnosis | ICD-10 | C31.8 | Malignant neoplasm of overlapping sites of accessory sinuses |
| Cancer Diagnosis | ICD-10 | C31.9 | Malignant neoplasm of accessory sinus, unspecified |
| Cancer Diagnosis | ICD-10 | C32.0 | Malignant neoplasm of glottis |
| Cancer Diagnosis | ICD-10 | C32.1 | Malignant neoplasm of supraglottis |
| Cancer Diagnosis | ICD-10 | C32.2 | Malignant neoplasm of subglottis |
| Cancer Diagnosis | ICD-10 | C32.3 | Malignant neoplasm of laryngeal cartilage |
| Cancer Diagnosis | ICD-10 | C32.8 | Malignant neoplasm of overlapping sites of larynx |
| Cancer Diagnosis | ICD-10 | C32.9 | Malignant neoplasm of larynx, unspecified |
| Cancer Diagnosis | ICD-10 | C33 | Malignant neoplasm of trachea |
| Cancer Diagnosis | ICD-10 | C34.00 | Malignant neoplasm of unspecified main bronchus |
| Cancer Diagnosis | ICD-10 | C34.01 | Malignant neoplasm of right main bronchus |
| Cancer Diagnosis | ICD-10 | C34.02 | Malignant neoplasm of left main bronchus |
| Cancer Diagnosis | ICD-10 | C34.10 | Malignant neoplasm of upper lobe, unspecified bronchus or lung |
| Cancer Diagnosis | ICD-10 | C34.11 | Malignant neoplasm of upper lobe, right bronchus or lung |
| Cancer Diagnosis | ICD-10 | C34.12 | Malignant neoplasm of upper lobe, left bronchus or lung |
| Cancer Diagnosis | ICD-10 | C34.2 | Malignant neoplasm of middle lobe, bronchus or lung |
| Cancer Diagnosis | ICD-10 | C34.30 | Malignant neoplasm of lower lobe, unspecified bronchus or lung |
| Cancer Diagnosis | ICD-10 | C34.31 | Malignant neoplasm of lower lobe, right bronchus or lung |
| Cancer Diagnosis | ICD-10 | C34.32 | Malignant neoplasm of lower lobe, left bronchus or lung |
| Cancer Diagnosis | ICD-10 | C34.80 | Malignant neoplasm of overlapping sites of unspecified bronchus and lung |
| Cancer Diagnosis | ICD-10 | C34.81 | Malignant neoplasm of overlapping sites of right bronchus and lung |
| Cancer Diagnosis | ICD-10 | C34.82 | Malignant neoplasm of overlapping sites of left bronchus and lung |
| Cancer Diagnosis | ICD-10 | C34.90 | Malignant neoplasm of unspecified part of unspecified bronchus or lung |
| Cancer Diagnosis | ICD-10 | C34.91 | Malignant neoplasm of unspecified part of right bronchus or lung |
| Cancer Diagnosis | ICD-10 | C34.92 | Malignant neoplasm of unspecified part of left bronchus or lung |
| Cancer Diagnosis | ICD-10 | C37 | Malignant neoplasm of thymus |
| Cancer Diagnosis | ICD-10 | C38.0 | Malignant neoplasm of heart |
| Cancer Diagnosis | ICD-10 | C38.1 | Malignant neoplasm of anterior mediastinum |
| Cancer Diagnosis | ICD-10 | C38.2 | Malignant neoplasm of posterior mediastinum |
| Cancer Diagnosis | ICD-10 | C38.3 | Malignant neoplasm of mediastinum, part unspecified |
| Cancer Diagnosis | ICD-10 | C38.4 | Malignant neoplasm of pleura |
| Cancer Diagnosis | ICD-10 | C38.8 | Malignant neoplasm of overlapping sites of heart, mediastinum and pleura |
| Cancer Diagnosis | ICD-10 | C39.0 | Malignant neoplasm of upper respiratory tract, part unspecified |
| Cancer Diagnosis | ICD-10 | C39.9 | Malignant neoplasm of lower respiratory tract, part unspecified |
| Cancer Diagnosis | ICD-10 | C40.00 | Malignant neoplasm of scapula and long bones of unspecified upper limb |
| Cancer Diagnosis | ICD-10 | C40.01 | Malignant neoplasm of scapula and long bones of right upper limb |
| Cancer Diagnosis | ICD-10 | C40.02 | Malignant neoplasm of scapula and long bones of left upper limb |
| Cancer Diagnosis | ICD-10 | C40.10 | Malignant neoplasm of short bones of unspecified upper limb |
| Cancer Diagnosis | ICD-10 | C40.11 | Malignant neoplasm of short bones of right upper limb |
| Cancer Diagnosis | ICD-10 | C40.12 | Malignant neoplasm of short bones of left upper limb |
| Cancer Diagnosis | ICD-10 | C40.20 | Malignant neoplasm of long bones of unspecified lower limb |
| Cancer Diagnosis | ICD-10 | C40.21 | Malignant neoplasm of long bones of right lower limb |
| Cancer Diagnosis | ICD-10 | C40.22 | Malignant neoplasm of long bones of left lower limb |
| Cancer Diagnosis | ICD-10 | C40.30 | Malignant neoplasm of short bones of unspecified lower limb |
| Cancer Diagnosis | ICD-10 | C40.31 | Malignant neoplasm of short bones of right lower limb |
| Cancer Diagnosis | ICD-10 | C40.32 | Malignant neoplasm of short bones of left lower limb |
| Cancer Diagnosis | ICD-10 | C40.80 | Malignant neoplasm of overlapping sites of bone and articular cartilage of unspecified limb |
| Cancer Diagnosis | ICD-10 | C40.81 | Malignant neoplasm of overlapping sites of bone and articular cartilage of right limb |
| Cancer Diagnosis | ICD-10 | C40.82 | Malignant neoplasm of overlapping sites of bone and articular cartilage of left limb |
| Cancer Diagnosis | ICD-10 | C40.90 | Malignant neoplasm of unspecified bones and articular cartilage of unspecified limb |
| Cancer Diagnosis | ICD-10 | C40.91 | Malignant neoplasm of unspecified bones and articular cartilage of right limb |
| Cancer Diagnosis | ICD-10 | C40.92 | Malignant neoplasm of unspecified bones and articular cartilage of left limb |
| Cancer Diagnosis | ICD-10 | C41.0 | Malignant neoplasm of bones of skull and face |
| Cancer Diagnosis | ICD-10 | C41.1 | Malignant neoplasm of mandible |
| Cancer Diagnosis | ICD-10 | C41.2 | Malignant neoplasm of vertebral column |
| Cancer Diagnosis | ICD-10 | C41.3 | Malignant neoplasm of ribs, sternum and clavicle |
| Cancer Diagnosis | ICD-10 | C41.4 | Malignant neoplasm of pelvic bones, sacrum and coccyx |
| Cancer Diagnosis | ICD-10 | C41.9 | Malignant neoplasm of bone and articular cartilage, unspecified |
| Cancer Diagnosis | ICD-10 | C43.0 | Malignant melanoma of lip |
| Cancer Diagnosis | ICD-10 | C43.10 | Malignant melanoma of unspecified eyelid, including canthus |
| Cancer Diagnosis | ICD-10 | C43.111 | Malignant melanoma of right upper eyelid, including canthus |
| Cancer Diagnosis | ICD-10 | C43.112 | Malignant melanoma of right lower eyelid, including canthus |
| Cancer Diagnosis | ICD-10 | C43.121 | Malignant melanoma of left upper eyelid, including canthus |
| Cancer Diagnosis | ICD-10 | C43.122 | Malignant melanoma of left lower eyelid, including canthus |
| Cancer Diagnosis | ICD-10 | C43.20 | Malignant melanoma of unspecified ear and external auricular canal |
| Cancer Diagnosis | ICD-10 | C43.21 | Malignant melanoma of right ear and external auricular canal |
| Cancer Diagnosis | ICD-10 | C43.22 | Malignant melanoma of left ear and external auricular canal |
| Cancer Diagnosis | ICD-10 | C43.30 | Malignant melanoma of unspecified part of face |
| Cancer Diagnosis | ICD-10 | C43.31 | Malignant melanoma of nose |
| Cancer Diagnosis | ICD-10 | C43.39 | Malignant melanoma of other parts of face |
| Cancer Diagnosis | ICD-10 | C43.4 | Malignant melanoma of scalp and neck |
| Cancer Diagnosis | ICD-10 | C43.51 | Malignant melanoma of anal skin |
| Cancer Diagnosis | ICD-10 | C43.52 | Malignant melanoma of skin of breast |
| Cancer Diagnosis | ICD-10 | C43.59 | Malignant melanoma of other part of trunk |
| Cancer Diagnosis | ICD-10 | C43.60 | Malignant melanoma of unspecified upper limb, including shoulder |
| Cancer Diagnosis | ICD-10 | C43.61 | Malignant melanoma of right upper limb, including shoulder |
| Cancer Diagnosis | ICD-10 | C43.62 | Malignant melanoma of left upper limb, including shoulder |
| Cancer Diagnosis | ICD-10 | C43.70 | Malignant melanoma of unspecified lower limb, including hip |
| Cancer Diagnosis | ICD-10 | C43.71 | Malignant melanoma of right lower limb, including hip |
| Cancer Diagnosis | ICD-10 | C43.72 | Malignant melanoma of left lower limb, including hip |
| Cancer Diagnosis | ICD-10 | C43.8 | Malignant melanoma of overlapping sites of skin |
| Cancer Diagnosis | ICD-10 | C43.9 | Malignant melanoma of skin, unspecified |
| Cancer Diagnosis | ICD-10 | C44.00 | Unspecified malignant neoplasm of skin of lip |
| Cancer Diagnosis | ICD-10 | C44.01 | Basal cell carcinoma of skin of lip |
| Cancer Diagnosis | ICD-10 | C44.02 | Squamous cell carcinoma of skin of lip |
| Cancer Diagnosis | ICD-10 | C44.09 | Other specified malignant neoplasm of skin of lip |
| Cancer Diagnosis | ICD-10 | C44.101 | Unspecified malignant neoplasm of skin of unspecified eyelid, including canthus |
| Cancer Diagnosis | ICD-10 | C44.1021 | Unspecified malignant neoplasm of skin of right upper eyelid, including canthus |
| Cancer Diagnosis | ICD-10 | C44.1022 | Unspecified malignant neoplasm of skin of right lower eyelid, including canthus |
| Cancer Diagnosis | ICD-10 | C44.1091 | Unspecified malignant neoplasm of skin of left upper eyelid, including canthus |
| Cancer Diagnosis | ICD-10 | C44.1092 | Unspecified malignant neoplasm of skin of left lower eyelid, including canthus |
| Cancer Diagnosis | ICD-10 | C44.111 | Basal cell carcinoma of skin of unspecified eyelid, including canthus |
| Cancer Diagnosis | ICD-10 | C44.1121 | Basal cell carcinoma of skin of right upper eyelid, including canthus |
| Cancer Diagnosis | ICD-10 | C44.1122 | Basal cell carcinoma of skin of right lower eyelid, including canthus |
| Cancer Diagnosis | ICD-10 | C44.1191 | Basal cell carcinoma of skin of left upper eyelid, including canthus |
| Cancer Diagnosis | ICD-10 | C44.1192 | Basal cell carcinoma of skin of left lower eyelid, including canthus |
| Cancer Diagnosis | ICD-10 | C44.121 | Squamous cell carcinoma of skin of unspecified eyelid, including canthus |
| Cancer Diagnosis | ICD-10 | C44.1221 | Squamous cell carcinoma of skin of right upper eyelid, including canthus |
| Cancer Diagnosis | ICD-10 | C44.1222 | Squamous cell carcinoma of skin of right lower eyelid, including canthus |
| Cancer Diagnosis | ICD-10 | C44.1291 | Squamous cell carcinoma of skin of left upper eyelid, including canthus |
| Cancer Diagnosis | ICD-10 | C44.1292 | Squamous cell carcinoma of skin of left lower eyelid, including canthus |
| Cancer Diagnosis | ICD-10 | C44.131 | Sebaceous cell carcinoma of skin of unspecified eyelid, including canthus |
| Cancer Diagnosis | ICD-10 | C44.1321 | Sebaceous cell carcinoma of skin of right upper eyelid, including canthus |
| Cancer Diagnosis | ICD-10 | C44.1322 | Sebaceous cell carcinoma of skin of right lower eyelid, including canthus |
| Cancer Diagnosis | ICD-10 | C44.1391 | Sebaceous cell carcinoma of skin of left upper eyelid, including canthus |
| Cancer Diagnosis | ICD-10 | C44.1392 | Sebaceous cell carcinoma of skin of left lower eyelid, including canthus |
| Cancer Diagnosis | ICD-10 | C44.191 | Other specified malignant neoplasm of skin of unspecified eyelid, including canthus |
| Cancer Diagnosis | ICD-10 | C44.1921 | Other specified malignant neoplasm of skin of right upper eyelid, including canthus |
| Cancer Diagnosis | ICD-10 | C44.1922 | Other specified malignant neoplasm of skin of right lower eyelid, including canthus |
| Cancer Diagnosis | ICD-10 | C44.1991 | Other specified malignant neoplasm of skin of left upper eyelid, including canthus |
| Cancer Diagnosis | ICD-10 | C44.1992 | Other specified malignant neoplasm of skin of left lower eyelid, including canthus |
| Cancer Diagnosis | ICD-10 | C44.201 | Unspecified malignant neoplasm of skin of unspecified ear and external auricular canal |
| Cancer Diagnosis | ICD-10 | C44.202 | Unspecified malignant neoplasm of skin of right ear and external auricular canal |
| Cancer Diagnosis | ICD-10 | C44.209 | Unspecified malignant neoplasm of skin of left ear and external auricular canal |
| Cancer Diagnosis | ICD-10 | C44.211 | Basal cell carcinoma of skin of unspecified ear and external auricular canal |
| Cancer Diagnosis | ICD-10 | C44.212 | Basal cell carcinoma of skin of right ear and external auricular canal |
| Cancer Diagnosis | ICD-10 | C44.219 | Basal cell carcinoma of skin of left ear and external auricular canal |
| Cancer Diagnosis | ICD-10 | C44.221 | Squamous cell carcinoma of skin of unspecified ear and external auricular canal |
| Cancer Diagnosis | ICD-10 | C44.222 | Squamous cell carcinoma of skin of right ear and external auricular canal |
| Cancer Diagnosis | ICD-10 | C44.229 | Squamous cell carcinoma of skin of left ear and external auricular canal |
| Cancer Diagnosis | ICD-10 | C44.291 | Other specified malignant neoplasm of skin of unspecified ear and external auricular canal |
| Cancer Diagnosis | ICD-10 | C44.292 | Other specified malignant neoplasm of skin of right ear and external auricular canal |
| Cancer Diagnosis | ICD-10 | C44.299 | Other specified malignant neoplasm of skin of left ear and external auricular canal |
| Cancer Diagnosis | ICD-10 | C44.300 | Unspecified malignant neoplasm of skin of unspecified part of face |
| Cancer Diagnosis | ICD-10 | C44.301 | Unspecified malignant neoplasm of skin of nose |
| Cancer Diagnosis | ICD-10 | C44.309 | Unspecified malignant neoplasm of skin of other parts of face |
| Cancer Diagnosis | ICD-10 | C44.310 | Basal cell carcinoma of skin of unspecified parts of face |
| Cancer Diagnosis | ICD-10 | C44.311 | Basal cell carcinoma of skin of nose |
| Cancer Diagnosis | ICD-10 | C44.319 | Basal cell carcinoma of skin of other parts of face |
| Cancer Diagnosis | ICD-10 | C44.320 | Squamous cell carcinoma of skin of unspecified parts of face |
| Cancer Diagnosis | ICD-10 | C44.321 | Squamous cell carcinoma of skin of nose |
| Cancer Diagnosis | ICD-10 | C44.329 | Squamous cell carcinoma of skin of other parts of face |
| Cancer Diagnosis | ICD-10 | C44.390 | Other specified malignant neoplasm of skin of unspecified parts of face |
| Cancer Diagnosis | ICD-10 | C44.391 | Other specified malignant neoplasm of skin of nose |
| Cancer Diagnosis | ICD-10 | C44.399 | Other specified malignant neoplasm of skin of other parts of face |
| Cancer Diagnosis | ICD-10 | C44.40 | Unspecified malignant neoplasm of skin of scalp and neck |
| Cancer Diagnosis | ICD-10 | C44.41 | Basal cell carcinoma of skin of scalp and neck |
| Cancer Diagnosis | ICD-10 | C44.42 | Squamous cell carcinoma of skin of scalp and neck |
| Cancer Diagnosis | ICD-10 | C44.49 | Other specified malignant neoplasm of skin of scalp and neck |
| Cancer Diagnosis | ICD-10 | C44.500 | Unspecified malignant neoplasm of anal skin |
| Cancer Diagnosis | ICD-10 | C44.501 | Unspecified malignant neoplasm of skin of breast |
| Cancer Diagnosis | ICD-10 | C44.509 | Unspecified malignant neoplasm of skin of other part of trunk |
| Cancer Diagnosis | ICD-10 | C44.510 | Basal cell carcinoma of anal skin |
| Cancer Diagnosis | ICD-10 | C44.511 | Basal cell carcinoma of skin of breast |
| Cancer Diagnosis | ICD-10 | C44.519 | Basal cell carcinoma of skin of other part of trunk |
| Cancer Diagnosis | ICD-10 | C44.520 | Squamous cell carcinoma of anal skin |
| Cancer Diagnosis | ICD-10 | C44.521 | Squamous cell carcinoma of skin of breast |
| Cancer Diagnosis | ICD-10 | C44.529 | Squamous cell carcinoma of skin of other part of trunk |
| Cancer Diagnosis | ICD-10 | C44.590 | Other specified malignant neoplasm of anal skin |
| Cancer Diagnosis | ICD-10 | C44.591 | Other specified malignant neoplasm of skin of breast |
| Cancer Diagnosis | ICD-10 | C44.599 | Other specified malignant neoplasm of skin of other part of trunk |
| Cancer Diagnosis | ICD-10 | C44.601 | Unspecified malignant neoplasm of skin of unspecified upper limb, including shoulder |
| Cancer Diagnosis | ICD-10 | C44.602 | Unspecified malignant neoplasm of skin of right upper limb, including shoulder |
| Cancer Diagnosis | ICD-10 | C44.609 | Unspecified malignant neoplasm of skin of left upper limb, including shoulder |
| Cancer Diagnosis | ICD-10 | C44.611 | Basal cell carcinoma of skin of unspecified upper limb, including shoulder |
| Cancer Diagnosis | ICD-10 | C44.612 | Basal cell carcinoma of skin of right upper limb, including shoulder |
| Cancer Diagnosis | ICD-10 | C44.619 | Basal cell carcinoma of skin of left upper limb, including shoulder |
| Cancer Diagnosis | ICD-10 | C44.621 | Squamous cell carcinoma of skin of unspecified upper limb, including shoulder |
| Cancer Diagnosis | ICD-10 | C44.622 | Squamous cell carcinoma of skin of right upper limb, including shoulder |
| Cancer Diagnosis | ICD-10 | C44.629 | Squamous cell carcinoma of skin of left upper limb, including shoulder |
| Cancer Diagnosis | ICD-10 | C44.691 | Other specified malignant neoplasm of skin of unspecified upper limb, including shoulder |
| Cancer Diagnosis | ICD-10 | C44.692 | Other specified malignant neoplasm of skin of right upper limb, including shoulder |
| Cancer Diagnosis | ICD-10 | C44.699 | Other specified malignant neoplasm of skin of left upper limb, including shoulder |
| Cancer Diagnosis | ICD-10 | C44.701 | Unspecified malignant neoplasm of skin of unspecified lower limb, including hip |
| Cancer Diagnosis | ICD-10 | C44.702 | Unspecified malignant neoplasm of skin of right lower limb, including hip |
| Cancer Diagnosis | ICD-10 | C44.709 | Unspecified malignant neoplasm of skin of left lower limb, including hip |
| Cancer Diagnosis | ICD-10 | C44.711 | Basal cell carcinoma of skin of unspecified lower limb, including hip |
| Cancer Diagnosis | ICD-10 | C44.712 | Basal cell carcinoma of skin of right lower limb, including hip |
| Cancer Diagnosis | ICD-10 | C44.719 | Basal cell carcinoma of skin of left lower limb, including hip |
| Cancer Diagnosis | ICD-10 | C44.721 | Squamous cell carcinoma of skin of unspecified lower limb, including hip |
| Cancer Diagnosis | ICD-10 | C44.722 | Squamous cell carcinoma of skin of right lower limb, including hip |
| Cancer Diagnosis | ICD-10 | C44.729 | Squamous cell carcinoma of skin of left lower limb, including hip |
| Cancer Diagnosis | ICD-10 | C44.791 | Other specified malignant neoplasm of skin of unspecified lower limb, including hip |
| Cancer Diagnosis | ICD-10 | C44.792 | Other specified malignant neoplasm of skin of right lower limb, including hip |
| Cancer Diagnosis | ICD-10 | C44.799 | Other specified malignant neoplasm of skin of left lower limb, including hip |
| Cancer Diagnosis | ICD-10 | C44.80 | Unspecified malignant neoplasm of overlapping sites of skin |
| Cancer Diagnosis | ICD-10 | C44.81 | Basal cell carcinoma of overlapping sites of skin |
| Cancer Diagnosis | ICD-10 | C44.82 | Squamous cell carcinoma of overlapping sites of skin |
| Cancer Diagnosis | ICD-10 | C44.89 | Other specified malignant neoplasm of overlapping sites of skin |
| Cancer Diagnosis | ICD-10 | C44.90 | Unspecified malignant neoplasm of skin, unspecified |
| Cancer Diagnosis | ICD-10 | C44.91 | Basal cell carcinoma of skin, unspecified |
| Cancer Diagnosis | ICD-10 | C44.92 | Squamous cell carcinoma of skin, unspecified |
| Cancer Diagnosis | ICD-10 | C44.99 | Other specified malignant neoplasm of skin, unspecified |
| Cancer Diagnosis | ICD-10 | C45.0 | Mesothelioma of pleura |
| Cancer Diagnosis | ICD-10 | C45.1 | Mesothelioma of peritoneum |
| Cancer Diagnosis | ICD-10 | C45.2 | Mesothelioma of pericardium |
| Cancer Diagnosis | ICD-10 | C45.7 | Mesothelioma of other sites |
| Cancer Diagnosis | ICD-10 | C45.9 | Mesothelioma, unspecified |
| Cancer Diagnosis | ICD-10 | C46.0 | Kaposi's sarcoma of skin |
| Cancer Diagnosis | ICD-10 | C46.1 | Kaposi's sarcoma of soft tissue |
| Cancer Diagnosis | ICD-10 | C46.2 | Kaposi's sarcoma of palate |
| Cancer Diagnosis | ICD-10 | C46.3 | Kaposi's sarcoma of lymph nodes |
| Cancer Diagnosis | ICD-10 | C46.4 | Kaposi's sarcoma of gastrointestinal sites |
| Cancer Diagnosis | ICD-10 | C46.50 | Kaposi's sarcoma of unspecified lung |
| Cancer Diagnosis | ICD-10 | C46.51 | Kaposi's sarcoma of right lung |
| Cancer Diagnosis | ICD-10 | C46.52 | Kaposi's sarcoma of left lung |
| Cancer Diagnosis | ICD-10 | C46.7 | Kaposi's sarcoma of other sites |
| Cancer Diagnosis | ICD-10 | C46.9 | Kaposi's sarcoma, unspecified |
| Cancer Diagnosis | ICD-10 | C47.0 | Malignant neoplasm of peripheral nerves of head, face and neck |
| Cancer Diagnosis | ICD-10 | C47.10 | Malignant neoplasm of peripheral nerves of unspecified upper limb, including shoulder |
| Cancer Diagnosis | ICD-10 | C47.11 | Malignant neoplasm of peripheral nerves of right upper limb, including shoulder |
| Cancer Diagnosis | ICD-10 | C47.12 | Malignant neoplasm of peripheral nerves of left upper limb, including shoulder |
| Cancer Diagnosis | ICD-10 | C47.20 | Malignant neoplasm of peripheral nerves of unspecified lower limb, including hip |
| Cancer Diagnosis | ICD-10 | C47.21 | Malignant neoplasm of peripheral nerves of right lower limb, including hip |
| Cancer Diagnosis | ICD-10 | C47.22 | Malignant neoplasm of peripheral nerves of left lower limb, including hip |
| Cancer Diagnosis | ICD-10 | C47.3 | Malignant neoplasm of peripheral nerves of thorax |
| Cancer Diagnosis | ICD-10 | C47.4 | Malignant neoplasm of peripheral nerves of abdomen |
| Cancer Diagnosis | ICD-10 | C47.5 | Malignant neoplasm of peripheral nerves of pelvis |
| Cancer Diagnosis | ICD-10 | C47.6 | Malignant neoplasm of peripheral nerves of trunk, unspecified |
| Cancer Diagnosis | ICD-10 | C47.8 | Malignant neoplasm of overlapping sites of peripheral nerves and autonomic nervous system |
| Cancer Diagnosis | ICD-10 | C47.9 | Malignant neoplasm of peripheral nerves and autonomic nervous system, unspecified |
| Cancer Diagnosis | ICD-10 | C48.0 | Malignant neoplasm of retroperitoneum |
| Cancer Diagnosis | ICD-10 | C48.1 | Malignant neoplasm of specified parts of peritoneum |
| Cancer Diagnosis | ICD-10 | C48.2 | Malignant neoplasm of peritoneum, unspecified |
| Cancer Diagnosis | ICD-10 | C48.8 | Malignant neoplasm of overlapping sites of retroperitoneum and peritoneum |
| Cancer Diagnosis | ICD-10 | C49.0 | Malignant neoplasm of connective and soft tissue of head, face and neck |
| Cancer Diagnosis | ICD-10 | C49.10 | Malignant neoplasm of connective and soft tissue of unspecified upper limb, including shoulder |
| Cancer Diagnosis | ICD-10 | C49.11 | Malignant neoplasm of connective and soft tissue of right upper limb, including shoulder |
| Cancer Diagnosis | ICD-10 | C49.12 | Malignant neoplasm of connective and soft tissue of left upper limb, including shoulder |
| Cancer Diagnosis | ICD-10 | C49.20 | Malignant neoplasm of connective and soft tissue of unspecified lower limb, including hip |
| Cancer Diagnosis | ICD-10 | C49.21 | Malignant neoplasm of connective and soft tissue of right lower limb, including hip |
| Cancer Diagnosis | ICD-10 | C49.22 | Malignant neoplasm of connective and soft tissue of left lower limb, including hip |
| Cancer Diagnosis | ICD-10 | C49.3 | Malignant neoplasm of connective and soft tissue of thorax |
| Cancer Diagnosis | ICD-10 | C49.4 | Malignant neoplasm of connective and soft tissue of abdomen |
| Cancer Diagnosis | ICD-10 | C49.5 | Malignant neoplasm of connective and soft tissue of pelvis |
| Cancer Diagnosis | ICD-10 | C49.6 | Malignant neoplasm of connective and soft tissue of trunk, unspecified |
| Cancer Diagnosis | ICD-10 | C49.8 | Malignant neoplasm of overlapping sites of connective and soft tissue |
| Cancer Diagnosis | ICD-10 | C49.9 | Malignant neoplasm of connective and soft tissue, unspecified |
| Cancer Diagnosis | ICD-10 | C49.A0 | Gastrointestinal stromal tumor, unspecified site |
| Cancer Diagnosis | ICD-10 | C49.A1 | Gastrointestinal stromal tumor of esophagus |
| Cancer Diagnosis | ICD-10 | C49.A2 | Gastrointestinal stromal tumor of stomach |
| Cancer Diagnosis | ICD-10 | C49.A3 | Gastrointestinal stromal tumor of small intestine |
| Cancer Diagnosis | ICD-10 | C49.A4 | Gastrointestinal stromal tumor of large intestine |
| Cancer Diagnosis | ICD-10 | C49.A5 | Gastrointestinal stromal tumor of rectum |
| Cancer Diagnosis | ICD-10 | C49.A9 | Gastrointestinal stromal tumor of other sites |
| Cancer Diagnosis | ICD-10 | C4A.0 | Merkel cell carcinoma of lip |
| Cancer Diagnosis | ICD-10 | C4A.10 | Merkel cell carcinoma of unspecified eyelid, including canthus |
| Cancer Diagnosis | ICD-10 | C4A.111 | Merkel cell carcinoma of right upper eyelid, including canthus |
| Cancer Diagnosis | ICD-10 | C4A.112 | Merkel cell carcinoma of right lower eyelid, including canthus |
| Cancer Diagnosis | ICD-10 | C4A.121 | Merkel cell carcinoma of left upper eyelid, including canthus |
| Cancer Diagnosis | ICD-10 | C4A.122 | Merkel cell carcinoma of left lower eyelid, including canthus |
| Cancer Diagnosis | ICD-10 | C4A.20 | Merkel cell carcinoma of unspecified ear and external auricular canal |
| Cancer Diagnosis | ICD-10 | C4A.21 | Merkel cell carcinoma of right ear and external auricular canal |
| Cancer Diagnosis | ICD-10 | C4A.22 | Merkel cell carcinoma of left ear and external auricular canal |
| Cancer Diagnosis | ICD-10 | C4A.30 | Merkel cell carcinoma of unspecified part of face |
| Cancer Diagnosis | ICD-10 | C4A.31 | Merkel cell carcinoma of nose |
| Cancer Diagnosis | ICD-10 | C4A.39 | Merkel cell carcinoma of other parts of face |
| Cancer Diagnosis | ICD-10 | C4A.4 | Merkel cell carcinoma of scalp and neck |
| Cancer Diagnosis | ICD-10 | C4A.51 | Merkel cell carcinoma of anal skin |
| Cancer Diagnosis | ICD-10 | C4A.52 | Merkel cell carcinoma of skin of breast |
| Cancer Diagnosis | ICD-10 | C4A.59 | Merkel cell carcinoma of other part of trunk |
| Cancer Diagnosis | ICD-10 | C4A.60 | Merkel cell carcinoma of unspecified upper limb, including shoulder |
| Cancer Diagnosis | ICD-10 | C4A.61 | Merkel cell carcinoma of right upper limb, including shoulder |
| Cancer Diagnosis | ICD-10 | C4A.62 | Merkel cell carcinoma of left upper limb, including shoulder |
| Cancer Diagnosis | ICD-10 | C4A.70 | Merkel cell carcinoma of unspecified lower limb, including hip |
| Cancer Diagnosis | ICD-10 | C4A.71 | Merkel cell carcinoma of right lower limb, including hip |
| Cancer Diagnosis | ICD-10 | C4A.72 | Merkel cell carcinoma of left lower limb, including hip |
| Cancer Diagnosis | ICD-10 | C4A.8 | Merkel cell carcinoma of overlapping sites |
| Cancer Diagnosis | ICD-10 | C4A.9 | Merkel cell carcinoma, unspecified |
| Cancer Diagnosis | ICD-10 | C50.011 | Malignant neoplasm of nipple and areola, right female breast |
| Cancer Diagnosis | ICD-10 | C50.012 | Malignant neoplasm of nipple and areola, left female breast |
| Cancer Diagnosis | ICD-10 | C50.019 | Malignant neoplasm of nipple and areola, unspecified female breast |
| Cancer Diagnosis | ICD-10 | C50.021 | Malignant neoplasm of nipple and areola, right male breast |
| Cancer Diagnosis | ICD-10 | C50.022 | Malignant neoplasm of nipple and areola, left male breast |
| Cancer Diagnosis | ICD-10 | C50.029 | Malignant neoplasm of nipple and areola, unspecified male breast |
| Cancer Diagnosis | ICD-10 | C50.111 | Malignant neoplasm of central portion of right female breast |
| Cancer Diagnosis | ICD-10 | C50.112 | Malignant neoplasm of central portion of left female breast |
| Cancer Diagnosis | ICD-10 | C50.119 | Malignant neoplasm of central portion of unspecified female breast |
| Cancer Diagnosis | ICD-10 | C50.121 | Malignant neoplasm of central portion of right male breast |
| Cancer Diagnosis | ICD-10 | C50.122 | Malignant neoplasm of central portion of left male breast |
| Cancer Diagnosis | ICD-10 | C50.129 | Malignant neoplasm of central portion of unspecified male breast |
| Cancer Diagnosis | ICD-10 | C50.211 | Malignant neoplasm of upper-inner quadrant of right female breast |
| Cancer Diagnosis | ICD-10 | C50.212 | Malignant neoplasm of upper-inner quadrant of left female breast |
| Cancer Diagnosis | ICD-10 | C50.219 | Malignant neoplasm of upper-inner quadrant of unspecified female breast |
| Cancer Diagnosis | ICD-10 | C50.221 | Malignant neoplasm of upper-inner quadrant of right male breast |
| Cancer Diagnosis | ICD-10 | C50.222 | Malignant neoplasm of upper-inner quadrant of left male breast |
| Cancer Diagnosis | ICD-10 | C50.229 | Malignant neoplasm of upper-inner quadrant of unspecified male breast |
| Cancer Diagnosis | ICD-10 | C50.311 | Malignant neoplasm of lower-inner quadrant of right female breast |
| Cancer Diagnosis | ICD-10 | C50.312 | Malignant neoplasm of lower-inner quadrant of left female breast |
| Cancer Diagnosis | ICD-10 | C50.319 | Malignant neoplasm of lower-inner quadrant of unspecified female breast |
| Cancer Diagnosis | ICD-10 | C50.321 | Malignant neoplasm of lower-inner quadrant of right male breast |
| Cancer Diagnosis | ICD-10 | C50.322 | Malignant neoplasm of lower-inner quadrant of left male breast |
| Cancer Diagnosis | ICD-10 | C50.329 | Malignant neoplasm of lower-inner quadrant of unspecified male breast |
| Cancer Diagnosis | ICD-10 | C50.411 | Malignant neoplasm of upper-outer quadrant of right female breast |
| Cancer Diagnosis | ICD-10 | C50.412 | Malignant neoplasm of upper-outer quadrant of left female breast |
| Cancer Diagnosis | ICD-10 | C50.419 | Malignant neoplasm of upper-outer quadrant of unspecified female breast |
| Cancer Diagnosis | ICD-10 | C50.421 | Malignant neoplasm of upper-outer quadrant of right male breast |
| Cancer Diagnosis | ICD-10 | C50.422 | Malignant neoplasm of upper-outer quadrant of left male breast |
| Cancer Diagnosis | ICD-10 | C50.429 | Malignant neoplasm of upper-outer quadrant of unspecified male breast |
| Cancer Diagnosis | ICD-10 | C50.511 | Malignant neoplasm of lower-outer quadrant of right female breast |
| Cancer Diagnosis | ICD-10 | C50.512 | Malignant neoplasm of lower-outer quadrant of left female breast |
| Cancer Diagnosis | ICD-10 | C50.519 | Malignant neoplasm of lower-outer quadrant of unspecified female breast |
| Cancer Diagnosis | ICD-10 | C50.521 | Malignant neoplasm of lower-outer quadrant of right male breast |
| Cancer Diagnosis | ICD-10 | C50.522 | Malignant neoplasm of lower-outer quadrant of left male breast |
| Cancer Diagnosis | ICD-10 | C50.529 | Malignant neoplasm of lower-outer quadrant of unspecified male breast |
| Cancer Diagnosis | ICD-10 | C50.611 | Malignant neoplasm of axillary tail of right female breast |
| Cancer Diagnosis | ICD-10 | C50.612 | Malignant neoplasm of axillary tail of left female breast |
| Cancer Diagnosis | ICD-10 | C50.619 | Malignant neoplasm of axillary tail of unspecified female breast |
| Cancer Diagnosis | ICD-10 | C50.621 | Malignant neoplasm of axillary tail of right male breast |
| Cancer Diagnosis | ICD-10 | C50.622 | Malignant neoplasm of axillary tail of left male breast |
| Cancer Diagnosis | ICD-10 | C50.629 | Malignant neoplasm of axillary tail of unspecified male breast |
| Cancer Diagnosis | ICD-10 | C50.811 | Malignant neoplasm of overlapping sites of right female breast |
| Cancer Diagnosis | ICD-10 | C50.812 | Malignant neoplasm of overlapping sites of left female breast |
| Cancer Diagnosis | ICD-10 | C50.819 | Malignant neoplasm of overlapping sites of unspecified female breast |
| Cancer Diagnosis | ICD-10 | C50.821 | Malignant neoplasm of overlapping sites of right male breast |
| Cancer Diagnosis | ICD-10 | C50.822 | Malignant neoplasm of overlapping sites of left male breast |
| Cancer Diagnosis | ICD-10 | C50.829 | Malignant neoplasm of overlapping sites of unspecified male breast |
| Cancer Diagnosis | ICD-10 | C50.911 | Malignant neoplasm of unspecified site of right female breast |
| Cancer Diagnosis | ICD-10 | C50.912 | Malignant neoplasm of unspecified site of left female breast |
| Cancer Diagnosis | ICD-10 | C50.919 | Malignant neoplasm of unspecified site of unspecified female breast |
| Cancer Diagnosis | ICD-10 | C50.921 | Malignant neoplasm of unspecified site of right male breast |
| Cancer Diagnosis | ICD-10 | C50.922 | Malignant neoplasm of unspecified site of left male breast |
| Cancer Diagnosis | ICD-10 | C50.929 | Malignant neoplasm of unspecified site of unspecified male breast |
| Cancer Diagnosis | ICD-10 | C51.0 | Malignant neoplasm of labium majus |
| Cancer Diagnosis | ICD-10 | C51.1 | Malignant neoplasm of labium minus |
| Cancer Diagnosis | ICD-10 | C51.2 | Malignant neoplasm of clitoris |
| Cancer Diagnosis | ICD-10 | C51.8 | Malignant neoplasm of overlapping sites of vulva |
| Cancer Diagnosis | ICD-10 | C51.9 | Malignant neoplasm of vulva, unspecified |
| Cancer Diagnosis | ICD-10 | C52 | Malignant neoplasm of vagina |
| Cancer Diagnosis | ICD-10 | C53.0 | Malignant neoplasm of endocervix |
| Cancer Diagnosis | ICD-10 | C53.1 | Malignant neoplasm of exocervix |
| Cancer Diagnosis | ICD-10 | C53.8 | Malignant neoplasm of overlapping sites of cervix uteri |
| Cancer Diagnosis | ICD-10 | C53.9 | Malignant neoplasm of cervix uteri, unspecified |
| Cancer Diagnosis | ICD-10 | C54.0 | Malignant neoplasm of isthmus uteri |
| Cancer Diagnosis | ICD-10 | C54.1 | Malignant neoplasm of endometrium |
| Cancer Diagnosis | ICD-10 | C54.2 | Malignant neoplasm of myometrium |
| Cancer Diagnosis | ICD-10 | C54.3 | Malignant neoplasm of fundus uteri |
| Cancer Diagnosis | ICD-10 | C54.8 | Malignant neoplasm of overlapping sites of corpus uteri |
| Cancer Diagnosis | ICD-10 | C54.9 | Malignant neoplasm of corpus uteri, unspecified |
| Cancer Diagnosis | ICD-10 | C55 | Malignant neoplasm of uterus, part unspecified |
| Cancer Diagnosis | ICD-10 | C56.1 | Malignant neoplasm of right ovary |
| Cancer Diagnosis | ICD-10 | C56.2 | Malignant neoplasm of left ovary |
| Cancer Diagnosis | ICD-10 | C56.9 | Malignant neoplasm of unspecified ovary |
| Cancer Diagnosis | ICD-10 | C57.00 | Malignant neoplasm of unspecified fallopian tube |
| Cancer Diagnosis | ICD-10 | C57.01 | Malignant neoplasm of right fallopian tube |
| Cancer Diagnosis | ICD-10 | C57.02 | Malignant neoplasm of left fallopian tube |
| Cancer Diagnosis | ICD-10 | C57.10 | Malignant neoplasm of unspecified broad ligament |
| Cancer Diagnosis | ICD-10 | C57.11 | Malignant neoplasm of right broad ligament |
| Cancer Diagnosis | ICD-10 | C57.12 | Malignant neoplasm of left broad ligament |
| Cancer Diagnosis | ICD-10 | C57.20 | Malignant neoplasm of unspecified round ligament |
| Cancer Diagnosis | ICD-10 | C57.21 | Malignant neoplasm of right round ligament |
| Cancer Diagnosis | ICD-10 | C57.22 | Malignant neoplasm of left round ligament |
| Cancer Diagnosis | ICD-10 | C57.3 | Malignant neoplasm of parametrium |
| Cancer Diagnosis | ICD-10 | C57.4 | Malignant neoplasm of uterine adnexa, unspecified |
| Cancer Diagnosis | ICD-10 | C57.7 | Malignant neoplasm of other specified female genital organs |
| Cancer Diagnosis | ICD-10 | C57.8 | Malignant neoplasm of overlapping sites of female genital organs |
| Cancer Diagnosis | ICD-10 | C57.9 | Malignant neoplasm of female genital organ, unspecified |
| Cancer Diagnosis | ICD-10 | C58 | Malignant neoplasm of placenta |
| Cancer Diagnosis | ICD-10 | C60.0 | Malignant neoplasm of prepuce |
| Cancer Diagnosis | ICD-10 | C60.1 | Malignant neoplasm of glans penis |
| Cancer Diagnosis | ICD-10 | C60.2 | Malignant neoplasm of body of penis |
| Cancer Diagnosis | ICD-10 | C60.8 | Malignant neoplasm of overlapping sites of penis |
| Cancer Diagnosis | ICD-10 | C60.9 | Malignant neoplasm of penis, unspecified |
| Cancer Diagnosis | ICD-10 | C61 | Malignant neoplasm of prostate |
| Cancer Diagnosis | ICD-10 | C62.00 | Malignant neoplasm of unspecified undescended testis |
| Cancer Diagnosis | ICD-10 | C62.01 | Malignant neoplasm of undescended right testis |
| Cancer Diagnosis | ICD-10 | C62.02 | Malignant neoplasm of undescended left testis |
| Cancer Diagnosis | ICD-10 | C62.10 | Malignant neoplasm of unspecified descended testis |
| Cancer Diagnosis | ICD-10 | C62.11 | Malignant neoplasm of descended right testis |
| Cancer Diagnosis | ICD-10 | C62.12 | Malignant neoplasm of descended left testis |
| Cancer Diagnosis | ICD-10 | C62.90 | Malignant neoplasm of unspecified testis, unspecified whether descended or undescended |
| Cancer Diagnosis | ICD-10 | C62.91 | Malignant neoplasm of right testis, unspecified whether descended or undescended |
| Cancer Diagnosis | ICD-10 | C62.92 | Malignant neoplasm of left testis, unspecified whether descended or undescended |
| Cancer Diagnosis | ICD-10 | C63.00 | Malignant neoplasm of unspecified epididymis |
| Cancer Diagnosis | ICD-10 | C63.01 | Malignant neoplasm of right epididymis |
| Cancer Diagnosis | ICD-10 | C63.02 | Malignant neoplasm of left epididymis |
| Cancer Diagnosis | ICD-10 | C63.10 | Malignant neoplasm of unspecified spermatic cord |
| Cancer Diagnosis | ICD-10 | C63.11 | Malignant neoplasm of right spermatic cord |
| Cancer Diagnosis | ICD-10 | C63.12 | Malignant neoplasm of left spermatic cord |
| Cancer Diagnosis | ICD-10 | C63.2 | Malignant neoplasm of scrotum |
| Cancer Diagnosis | ICD-10 | C63.7 | Malignant neoplasm of other specified male genital organs |
| Cancer Diagnosis | ICD-10 | C63.8 | Malignant neoplasm of overlapping sites of male genital organs |
| Cancer Diagnosis | ICD-10 | C63.9 | Malignant neoplasm of male genital organ, unspecified |
| Cancer Diagnosis | ICD-10 | C64.1 | Malignant neoplasm of right kidney, except renal pelvis |
| Cancer Diagnosis | ICD-10 | C64.2 | Malignant neoplasm of left kidney, except renal pelvis |
| Cancer Diagnosis | ICD-10 | C64.9 | Malignant neoplasm of unspecified kidney, except renal pelvis |
| Cancer Diagnosis | ICD-10 | C65.1 | Malignant neoplasm of right renal pelvis |
| Cancer Diagnosis | ICD-10 | C65.2 | Malignant neoplasm of left renal pelvis |
| Cancer Diagnosis | ICD-10 | C65.9 | Malignant neoplasm of unspecified renal pelvis |
| Cancer Diagnosis | ICD-10 | C66.1 | Malignant neoplasm of right ureter |
| Cancer Diagnosis | ICD-10 | C66.2 | Malignant neoplasm of left ureter |
| Cancer Diagnosis | ICD-10 | C66.9 | Malignant neoplasm of unspecified ureter |
| Cancer Diagnosis | ICD-10 | C67.0 | Malignant neoplasm of trigone of bladder |
| Cancer Diagnosis | ICD-10 | C67.1 | Malignant neoplasm of dome of bladder |
| Cancer Diagnosis | ICD-10 | C67.2 | Malignant neoplasm of lateral wall of bladder |
| Cancer Diagnosis | ICD-10 | C67.3 | Malignant neoplasm of anterior wall of bladder |
| Cancer Diagnosis | ICD-10 | C67.4 | Malignant neoplasm of posterior wall of bladder |
| Cancer Diagnosis | ICD-10 | C67.5 | Malignant neoplasm of bladder neck |
| Cancer Diagnosis | ICD-10 | C67.6 | Malignant neoplasm of ureteric orifice |
| Cancer Diagnosis | ICD-10 | C67.7 | Malignant neoplasm of urachus |
| Cancer Diagnosis | ICD-10 | C67.8 | Malignant neoplasm of overlapping sites of bladder |
| Cancer Diagnosis | ICD-10 | C67.9 | Malignant neoplasm of bladder, unspecified |
| Cancer Diagnosis | ICD-10 | C68.0 | Malignant neoplasm of urethra |
| Cancer Diagnosis | ICD-10 | C68.1 | Malignant neoplasm of paraurethral glands |
| Cancer Diagnosis | ICD-10 | C68.8 | Malignant neoplasm of overlapping sites of urinary organs |
| Cancer Diagnosis | ICD-10 | C68.9 | Malignant neoplasm of urinary organ, unspecified |
| Cancer Diagnosis | ICD-10 | C69.00 | Malignant neoplasm of unspecified conjunctiva |
| Cancer Diagnosis | ICD-10 | C69.01 | Malignant neoplasm of right conjunctiva |
| Cancer Diagnosis | ICD-10 | C69.02 | Malignant neoplasm of left conjunctiva |
| Cancer Diagnosis | ICD-10 | C69.10 | Malignant neoplasm of unspecified cornea |
| Cancer Diagnosis | ICD-10 | C69.11 | Malignant neoplasm of right cornea |
| Cancer Diagnosis | ICD-10 | C69.12 | Malignant neoplasm of left cornea |
| Cancer Diagnosis | ICD-10 | C69.20 | Malignant neoplasm of unspecified retina |
| Cancer Diagnosis | ICD-10 | C69.21 | Malignant neoplasm of right retina |
| Cancer Diagnosis | ICD-10 | C69.22 | Malignant neoplasm of left retina |
| Cancer Diagnosis | ICD-10 | C69.30 | Malignant neoplasm of unspecified choroid |
| Cancer Diagnosis | ICD-10 | C69.31 | Malignant neoplasm of right choroid |
| Cancer Diagnosis | ICD-10 | C69.32 | Malignant neoplasm of left choroid |
| Cancer Diagnosis | ICD-10 | C69.40 | Malignant neoplasm of unspecified ciliary body |
| Cancer Diagnosis | ICD-10 | C69.41 | Malignant neoplasm of right ciliary body |
| Cancer Diagnosis | ICD-10 | C69.42 | Malignant neoplasm of left ciliary body |
| Cancer Diagnosis | ICD-10 | C69.50 | Malignant neoplasm of unspecified lacrimal gland and duct |
| Cancer Diagnosis | ICD-10 | C69.51 | Malignant neoplasm of right lacrimal gland and duct |
| Cancer Diagnosis | ICD-10 | C69.52 | Malignant neoplasm of left lacrimal gland and duct |
| Cancer Diagnosis | ICD-10 | C69.60 | Malignant neoplasm of unspecified orbit |
| Cancer Diagnosis | ICD-10 | C69.61 | Malignant neoplasm of right orbit |
| Cancer Diagnosis | ICD-10 | C69.62 | Malignant neoplasm of left orbit |
| Cancer Diagnosis | ICD-10 | C69.80 | Malignant neoplasm of overlapping sites of unspecified eye and adnexa |
| Cancer Diagnosis | ICD-10 | C69.81 | Malignant neoplasm of overlapping sites of right eye and adnexa |
| Cancer Diagnosis | ICD-10 | C69.82 | Malignant neoplasm of overlapping sites of left eye and adnexa |
| Cancer Diagnosis | ICD-10 | C69.90 | Malignant neoplasm of unspecified site of unspecified eye |
| Cancer Diagnosis | ICD-10 | C69.91 | Malignant neoplasm of unspecified site of right eye |
| Cancer Diagnosis | ICD-10 | C69.92 | Malignant neoplasm of unspecified site of left eye |
| Cancer Diagnosis | ICD-10 | C70.0 | Malignant neoplasm of cerebral meninges |
| Cancer Diagnosis | ICD-10 | C70.1 | Malignant neoplasm of spinal meninges |
| Cancer Diagnosis | ICD-10 | C70.9 | Malignant neoplasm of meninges, unspecified |
| Cancer Diagnosis | ICD-10 | C71.0 | Malignant neoplasm of cerebrum, except lobes and ventricles |
| Cancer Diagnosis | ICD-10 | C71.1 | Malignant neoplasm of frontal lobe |
| Cancer Diagnosis | ICD-10 | C71.2 | Malignant neoplasm of temporal lobe |
| Cancer Diagnosis | ICD-10 | C71.3 | Malignant neoplasm of parietal lobe |
| Cancer Diagnosis | ICD-10 | C71.4 | Malignant neoplasm of occipital lobe |
| Cancer Diagnosis | ICD-10 | C71.5 | Malignant neoplasm of cerebral ventricle |
| Cancer Diagnosis | ICD-10 | C71.6 | Malignant neoplasm of cerebellum |
| Cancer Diagnosis | ICD-10 | C71.7 | Malignant neoplasm of brain stem |
| Cancer Diagnosis | ICD-10 | C71.8 | Malignant neoplasm of overlapping sites of brain |
| Cancer Diagnosis | ICD-10 | C71.9 | Malignant neoplasm of brain, unspecified |
| Cancer Diagnosis | ICD-10 | C72.0 | Malignant neoplasm of spinal cord |
| Cancer Diagnosis | ICD-10 | C72.1 | Malignant neoplasm of cauda equina |
| Cancer Diagnosis | ICD-10 | C72.20 | Malignant neoplasm of unspecified olfactory nerve |
| Cancer Diagnosis | ICD-10 | C72.21 | Malignant neoplasm of right olfactory nerve |
| Cancer Diagnosis | ICD-10 | C72.22 | Malignant neoplasm of left olfactory nerve |
| Cancer Diagnosis | ICD-10 | C72.30 | Malignant neoplasm of unspecified optic nerve |
| Cancer Diagnosis | ICD-10 | C72.31 | Malignant neoplasm of right optic nerve |
| Cancer Diagnosis | ICD-10 | C72.32 | Malignant neoplasm of left optic nerve |
| Cancer Diagnosis | ICD-10 | C72.40 | Malignant neoplasm of unspecified acoustic nerve |
| Cancer Diagnosis | ICD-10 | C72.41 | Malignant neoplasm of right acoustic nerve |
| Cancer Diagnosis | ICD-10 | C72.42 | Malignant neoplasm of left acoustic nerve |
| Cancer Diagnosis | ICD-10 | C72.50 | Malignant neoplasm of unspecified cranial nerve |
| Cancer Diagnosis | ICD-10 | C72.59 | Malignant neoplasm of other cranial nerves |
| Cancer Diagnosis | ICD-10 | C72.9 | Malignant neoplasm of central nervous system, unspecified |
| Cancer Diagnosis | ICD-10 | C73 | Malignant neoplasm of thyroid gland |
| Cancer Diagnosis | ICD-10 | C74.00 | Malignant neoplasm of cortex of unspecified adrenal gland |
| Cancer Diagnosis | ICD-10 | C74.01 | Malignant neoplasm of cortex of right adrenal gland |
| Cancer Diagnosis | ICD-10 | C74.02 | Malignant neoplasm of cortex of left adrenal gland |
| Cancer Diagnosis | ICD-10 | C74.10 | Malignant neoplasm of medulla of unspecified adrenal gland |
| Cancer Diagnosis | ICD-10 | C74.11 | Malignant neoplasm of medulla of right adrenal gland |
| Cancer Diagnosis | ICD-10 | C74.12 | Malignant neoplasm of medulla of left adrenal gland |
| Cancer Diagnosis | ICD-10 | C74.90 | Malignant neoplasm of unspecified part of unspecified adrenal gland |
| Cancer Diagnosis | ICD-10 | C74.91 | Malignant neoplasm of unspecified part of right adrenal gland |
| Cancer Diagnosis | ICD-10 | C74.92 | Malignant neoplasm of unspecified part of left adrenal gland |
| Cancer Diagnosis | ICD-10 | C75.0 | Malignant neoplasm of parathyroid gland |
| Cancer Diagnosis | ICD-10 | C75.1 | Malignant neoplasm of pituitary gland |
| Cancer Diagnosis | ICD-10 | C75.2 | Malignant neoplasm of craniopharyngeal duct |
| Cancer Diagnosis | ICD-10 | C75.3 | Malignant neoplasm of pineal gland |
| Cancer Diagnosis | ICD-10 | C75.4 | Malignant neoplasm of carotid body |
| Cancer Diagnosis | ICD-10 | C75.5 | Malignant neoplasm of aortic body and other paraganglia |
| Cancer Diagnosis | ICD-10 | C75.8 | Malignant neoplasm with pluriglandular involvement, unspecified |
| Cancer Diagnosis | ICD-10 | C75.9 | Malignant neoplasm of endocrine gland, unspecified |
| Cancer Diagnosis | ICD-10 | C76.0 | Malignant neoplasm of head, face and neck |
| Cancer Diagnosis | ICD-10 | C76.1 | Malignant neoplasm of thorax |
| Cancer Diagnosis | ICD-10 | C76.2 | Malignant neoplasm of abdomen |
| Cancer Diagnosis | ICD-10 | C76.3 | Malignant neoplasm of pelvis |
| Cancer Diagnosis | ICD-10 | C76.40 | Malignant neoplasm of unspecified upper limb |
| Cancer Diagnosis | ICD-10 | C76.41 | Malignant neoplasm of right upper limb |
| Cancer Diagnosis | ICD-10 | C76.42 | Malignant neoplasm of left upper limb |
| Cancer Diagnosis | ICD-10 | C76.50 | Malignant neoplasm of unspecified lower limb |
| Cancer Diagnosis | ICD-10 | C76.51 | Malignant neoplasm of right lower limb |
| Cancer Diagnosis | ICD-10 | C76.52 | Malignant neoplasm of left lower limb |
| Cancer Diagnosis | ICD-10 | C76.8 | Malignant neoplasm of other specified ill-defined sites |
| Cancer Diagnosis | ICD-10 | C77.0 | Secondary and unspecified malignant neoplasm of lymph nodes of head, face and neck |
| Cancer Diagnosis | ICD-10 | C77.1 | Secondary and unspecified malignant neoplasm of intrathoracic lymph nodes |
| Cancer Diagnosis | ICD-10 | C77.2 | Secondary and unspecified malignant neoplasm of intra-abdominal lymph nodes |
| Cancer Diagnosis | ICD-10 | C77.3 | Secondary and unspecified malignant neoplasm of axilla and upper limb lymph nodes |
| Cancer Diagnosis | ICD-10 | C77.4 | Secondary and unspecified malignant neoplasm of inguinal and lower limb lymph nodes |
| Cancer Diagnosis | ICD-10 | C77.5 | Secondary and unspecified malignant neoplasm of intrapelvic lymph nodes |
| Cancer Diagnosis | ICD-10 | C77.8 | Secondary and unspecified malignant neoplasm of lymph nodes of multiple regions |
| Cancer Diagnosis | ICD-10 | C77.9 | Secondary and unspecified malignant neoplasm of lymph node, unspecified |
| Cancer Diagnosis | ICD-10 | C78.00 | Secondary malignant neoplasm of unspecified lung |
| Cancer Diagnosis | ICD-10 | C78.01 | Secondary malignant neoplasm of right lung |
| Cancer Diagnosis | ICD-10 | C78.02 | Secondary malignant neoplasm of left lung |
| Cancer Diagnosis | ICD-10 | C78.1 | Secondary malignant neoplasm of mediastinum |
| Cancer Diagnosis | ICD-10 | C78.2 | Secondary malignant neoplasm of pleura |
| Cancer Diagnosis | ICD-10 | C78.30 | Secondary malignant neoplasm of unspecified respiratory organ |
| Cancer Diagnosis | ICD-10 | C78.39 | Secondary malignant neoplasm of other respiratory organs |
| Cancer Diagnosis | ICD-10 | C78.4 | Secondary malignant neoplasm of small intestine |
| Cancer Diagnosis | ICD-10 | C78.5 | Secondary malignant neoplasm of large intestine and rectum |
| Cancer Diagnosis | ICD-10 | C78.6 | Secondary malignant neoplasm of retroperitoneum and peritoneum |
| Cancer Diagnosis | ICD-10 | C78.7 | Secondary malignant neoplasm of liver and intrahepatic bile duct |
| Cancer Diagnosis | ICD-10 | C78.80 | Secondary malignant neoplasm of unspecified digestive organ |
| Cancer Diagnosis | ICD-10 | C78.89 | Secondary malignant neoplasm of other digestive organs |
| Cancer Diagnosis | ICD-10 | C79.00 | Secondary malignant neoplasm of unspecified kidney and renal pelvis |
| Cancer Diagnosis | ICD-10 | C79.01 | Secondary malignant neoplasm of right kidney and renal pelvis |
| Cancer Diagnosis | ICD-10 | C79.02 | Secondary malignant neoplasm of left kidney and renal pelvis |
| Cancer Diagnosis | ICD-10 | C79.10 | Secondary malignant neoplasm of unspecified urinary organs |
| Cancer Diagnosis | ICD-10 | C79.11 | Secondary malignant neoplasm of bladder |
| Cancer Diagnosis | ICD-10 | C79.19 | Secondary malignant neoplasm of other urinary organs |
| Cancer Diagnosis | ICD-10 | C79.2 | Secondary malignant neoplasm of skin |
| Cancer Diagnosis | ICD-10 | C79.31 | Secondary malignant neoplasm of brain |
| Cancer Diagnosis | ICD-10 | C79.32 | Secondary malignant neoplasm of cerebral meninges |
| Cancer Diagnosis | ICD-10 | C79.40 | Secondary malignant neoplasm of unspecified part of nervous system |
| Cancer Diagnosis | ICD-10 | C79.49 | Secondary malignant neoplasm of other parts of nervous system |
| Cancer Diagnosis | ICD-10 | C79.51 | Secondary malignant neoplasm of bone |
| Cancer Diagnosis | ICD-10 | C79.52 | Secondary malignant neoplasm of bone marrow |
| Cancer Diagnosis | ICD-10 | C79.60 | Secondary malignant neoplasm of unspecified ovary |
| Cancer Diagnosis | ICD-10 | C79.61 | Secondary malignant neoplasm of right ovary |
| Cancer Diagnosis | ICD-10 | C79.62 | Secondary malignant neoplasm of left ovary |
| Cancer Diagnosis | ICD-10 | C79.70 | Secondary malignant neoplasm of unspecified adrenal gland |
| Cancer Diagnosis | ICD-10 | C79.71 | Secondary malignant neoplasm of right adrenal gland |
| Cancer Diagnosis | ICD-10 | C79.72 | Secondary malignant neoplasm of left adrenal gland |
| Cancer Diagnosis | ICD-10 | C79.81 | Secondary malignant neoplasm of breast |
| Cancer Diagnosis | ICD-10 | C79.82 | Secondary malignant neoplasm of genital organs |
| Cancer Diagnosis | ICD-10 | C79.89 | Secondary malignant neoplasm of other specified sites |
| Cancer Diagnosis | ICD-10 | C79.9 | Secondary malignant neoplasm of unspecified site |
| Cancer Diagnosis | ICD-10 | C7A.00 | Malignant carcinoid tumor of unspecified site |
| Cancer Diagnosis | ICD-10 | C7A.010 | Malignant carcinoid tumor of the duodenum |
| Cancer Diagnosis | ICD-10 | C7A.011 | Malignant carcinoid tumor of the jejunum |
| Cancer Diagnosis | ICD-10 | C7A.012 | Malignant carcinoid tumor of the ileum |
| Cancer Diagnosis | ICD-10 | C7A.019 | Malignant carcinoid tumor of the small intestine, unspecified portion |
| Cancer Diagnosis | ICD-10 | C7A.020 | Malignant carcinoid tumor of the appendix |
| Cancer Diagnosis | ICD-10 | C7A.021 | Malignant carcinoid tumor of the cecum |
| Cancer Diagnosis | ICD-10 | C7A.022 | Malignant carcinoid tumor of the ascending colon |
| Cancer Diagnosis | ICD-10 | C7A.023 | Malignant carcinoid tumor of the transverse colon |
| Cancer Diagnosis | ICD-10 | C7A.024 | Malignant carcinoid tumor of the descending colon |
| Cancer Diagnosis | ICD-10 | C7A.025 | Malignant carcinoid tumor of the sigmoid colon |
| Cancer Diagnosis | ICD-10 | C7A.026 | Malignant carcinoid tumor of the rectum |
| Cancer Diagnosis | ICD-10 | C7A.029 | Malignant carcinoid tumor of the large intestine, unspecified portion |
| Cancer Diagnosis | ICD-10 | C7A.090 | Malignant carcinoid tumor of the bronchus and lung |
| Cancer Diagnosis | ICD-10 | C7A.091 | Malignant carcinoid tumor of the thymus |
| Cancer Diagnosis | ICD-10 | C7A.092 | Malignant carcinoid tumor of the stomach |
| Cancer Diagnosis | ICD-10 | C7A.093 | Malignant carcinoid tumor of the kidney |
| Cancer Diagnosis | ICD-10 | C7A.094 | Malignant carcinoid tumor of the foregut, unspecified |
| Cancer Diagnosis | ICD-10 | C7A.095 | Malignant carcinoid tumor of the midgut, unspecified |
| Cancer Diagnosis | ICD-10 | C7A.096 | Malignant carcinoid tumor of the hindgut, unspecified |
| Cancer Diagnosis | ICD-10 | C7A.098 | Malignant carcinoid tumors of other sites |
| Cancer Diagnosis | ICD-10 | C7A.1 | Malignant poorly differentiated neuroendocrine tumors |
| Cancer Diagnosis | ICD-10 | C7A.8 | Other malignant neuroendocrine tumors |
| Cancer Diagnosis | ICD-10 | C7B.00 | Secondary carcinoid tumors, unspecified site |
| Cancer Diagnosis | ICD-10 | C7B.01 | Secondary carcinoid tumors of distant lymph nodes |
| Cancer Diagnosis | ICD-10 | C7B.02 | Secondary carcinoid tumors of liver |
| Cancer Diagnosis | ICD-10 | C7B.03 | Secondary carcinoid tumors of bone |
| Cancer Diagnosis | ICD-10 | C7B.04 | Secondary carcinoid tumors of peritoneum |
| Cancer Diagnosis | ICD-10 | C7B.09 | Secondary carcinoid tumors of other sites |
| Cancer Diagnosis | ICD-10 | C7B.1 | Secondary Merkel cell carcinoma |
| Cancer Diagnosis | ICD-10 | C7B.8 | Other secondary neuroendocrine tumors |
| Cancer Diagnosis | ICD-10 | C80.0 | Disseminated malignant neoplasm, unspecified |
| Cancer Diagnosis | ICD-10 | C80.1 | Malignant (primary) neoplasm, unspecified |
| Cancer Diagnosis | ICD-10 | C80.2 | Malignant neoplasm associated with transplanted organ |
| Cancer Diagnosis | ICD-10 | C81.00 | Nodular lymphocyte predominant Hodgkin lymphoma, unspecified site |
| Cancer Diagnosis | ICD-10 | C81.01 | Nodular lymphocyte predominant Hodgkin lymphoma, lymph nodes of head, face, and neck |
| Cancer Diagnosis | ICD-10 | C81.02 | Nodular lymphocyte predominant Hodgkin lymphoma, intrathoracic lymph nodes |
| Cancer Diagnosis | ICD-10 | C81.03 | Nodular lymphocyte predominant Hodgkin lymphoma, intra-abdominal lymph nodes |
| Cancer Diagnosis | ICD-10 | C81.04 | Nodular lymphocyte predominant Hodgkin lymphoma, lymph nodes of axilla and upper limb |
| Cancer Diagnosis | ICD-10 | C81.05 | Nodular lymphocyte predominant Hodgkin lymphoma, lymph nodes of inguinal region and lower limb |
| Cancer Diagnosis | ICD-10 | C81.06 | Nodular lymphocyte predominant Hodgkin lymphoma, intrapelvic lymph nodes |
| Cancer Diagnosis | ICD-10 | C81.07 | Nodular lymphocyte predominant Hodgkin lymphoma, spleen |
| Cancer Diagnosis | ICD-10 | C81.08 | Nodular lymphocyte predominant Hodgkin lymphoma, lymph nodes of multiple sites |
| Cancer Diagnosis | ICD-10 | C81.09 | Nodular lymphocyte predominant Hodgkin lymphoma, extranodal and solid organ sites |
| Cancer Diagnosis | ICD-10 | C81.10 | Nodular sclerosis Hodgkin lymphoma, unspecified site |
| Cancer Diagnosis | ICD-10 | C81.11 | Nodular sclerosis Hodgkin lymphoma, lymph nodes of head, face, and neck |
| Cancer Diagnosis | ICD-10 | C81.12 | Nodular sclerosis Hodgkin lymphoma, intrathoracic lymph nodes |
| Cancer Diagnosis | ICD-10 | C81.13 | Nodular sclerosis Hodgkin lymphoma, intra-abdominal lymph nodes |
| Cancer Diagnosis | ICD-10 | C81.14 | Nodular sclerosis Hodgkin lymphoma, lymph nodes of axilla and upper limb |
| Cancer Diagnosis | ICD-10 | C81.15 | Nodular sclerosis Hodgkin lymphoma, lymph nodes of inguinal region and lower limb |
| Cancer Diagnosis | ICD-10 | C81.16 | Nodular sclerosis Hodgkin lymphoma, intrapelvic lymph nodes |
| Cancer Diagnosis | ICD-10 | C81.17 | Nodular sclerosis Hodgkin lymphoma, spleen |
| Cancer Diagnosis | ICD-10 | C81.18 | Nodular sclerosis Hodgkin lymphoma, lymph nodes of multiple sites |
| Cancer Diagnosis | ICD-10 | C81.19 | Nodular sclerosis Hodgkin lymphoma, extranodal and solid organ sites |
| Cancer Diagnosis | ICD-10 | C81.20 | Mixed cellularity Hodgkin lymphoma, unspecified site |
| Cancer Diagnosis | ICD-10 | C81.21 | Mixed cellularity Hodgkin lymphoma, lymph nodes of head, face, and neck |
| Cancer Diagnosis | ICD-10 | C81.22 | Mixed cellularity Hodgkin lymphoma, intrathoracic lymph nodes |
| Cancer Diagnosis | ICD-10 | C81.23 | Mixed cellularity Hodgkin lymphoma, intra-abdominal lymph nodes |
| Cancer Diagnosis | ICD-10 | C81.24 | Mixed cellularity Hodgkin lymphoma, lymph nodes of axilla and upper limb |
| Cancer Diagnosis | ICD-10 | C81.25 | Mixed cellularity Hodgkin lymphoma, lymph nodes of inguinal region and lower limb |
| Cancer Diagnosis | ICD-10 | C81.26 | Mixed cellularity Hodgkin lymphoma, intrapelvic lymph nodes |
| Cancer Diagnosis | ICD-10 | C81.27 | Mixed cellularity Hodgkin lymphoma, spleen |
| Cancer Diagnosis | ICD-10 | C81.28 | Mixed cellularity Hodgkin lymphoma, lymph nodes of multiple sites |
| Cancer Diagnosis | ICD-10 | C81.29 | Mixed cellularity Hodgkin lymphoma, extranodal and solid organ sites |
| Cancer Diagnosis | ICD-10 | C81.30 | Lymphocyte depleted Hodgkin lymphoma, unspecified site |
| Cancer Diagnosis | ICD-10 | C81.31 | Lymphocyte depleted Hodgkin lymphoma, lymph nodes of head, face, and neck |
| Cancer Diagnosis | ICD-10 | C81.32 | Lymphocyte depleted Hodgkin lymphoma, intrathoracic lymph nodes |
| Cancer Diagnosis | ICD-10 | C81.33 | Lymphocyte depleted Hodgkin lymphoma, intra-abdominal lymph nodes |
| Cancer Diagnosis | ICD-10 | C81.34 | Lymphocyte depleted Hodgkin lymphoma, lymph nodes of axilla and upper limb |
| Cancer Diagnosis | ICD-10 | C81.35 | Lymphocyte depleted Hodgkin lymphoma, lymph nodes of inguinal region and lower limb |
| Cancer Diagnosis | ICD-10 | C81.36 | Lymphocyte depleted Hodgkin lymphoma, intrapelvic lymph nodes |
| Cancer Diagnosis | ICD-10 | C81.37 | Lymphocyte depleted Hodgkin lymphoma, spleen |
| Cancer Diagnosis | ICD-10 | C81.38 | Lymphocyte depleted Hodgkin lymphoma, lymph nodes of multiple sites |
| Cancer Diagnosis | ICD-10 | C81.39 | Lymphocyte depleted Hodgkin lymphoma, extranodal and solid organ sites |
| Cancer Diagnosis | ICD-10 | C81.40 | Lymphocyte-rich Hodgkin lymphoma, unspecified site |
| Cancer Diagnosis | ICD-10 | C81.41 | Lymphocyte-rich Hodgkin lymphoma, lymph nodes of head, face, and neck |
| Cancer Diagnosis | ICD-10 | C81.42 | Lymphocyte-rich Hodgkin lymphoma, intrathoracic lymph nodes |
| Cancer Diagnosis | ICD-10 | C81.43 | Lymphocyte-rich Hodgkin lymphoma, intra-abdominal lymph nodes |
| Cancer Diagnosis | ICD-10 | C81.44 | Lymphocyte-rich Hodgkin lymphoma, lymph nodes of axilla and upper limb |
| Cancer Diagnosis | ICD-10 | C81.45 | Lymphocyte-rich Hodgkin lymphoma, lymph nodes of inguinal region and lower limb |
| Cancer Diagnosis | ICD-10 | C81.46 | Lymphocyte-rich Hodgkin lymphoma, intrapelvic lymph nodes |
| Cancer Diagnosis | ICD-10 | C81.47 | Lymphocyte-rich Hodgkin lymphoma, spleen |
| Cancer Diagnosis | ICD-10 | C81.48 | Lymphocyte-rich Hodgkin lymphoma, lymph nodes of multiple sites |
| Cancer Diagnosis | ICD-10 | C81.49 | Lymphocyte-rich Hodgkin lymphoma, extranodal and solid organ sites |
| Cancer Diagnosis | ICD-10 | C81.70 | Other Hodgkin lymphoma, unspecified site |
| Cancer Diagnosis | ICD-10 | C81.71 | Other Hodgkin lymphoma, lymph nodes of head, face, and neck |
| Cancer Diagnosis | ICD-10 | C81.72 | Other Hodgkin lymphoma, intrathoracic lymph nodes |
| Cancer Diagnosis | ICD-10 | C81.73 | Other Hodgkin lymphoma, intra-abdominal lymph nodes |
| Cancer Diagnosis | ICD-10 | C81.74 | Other Hodgkin lymphoma, lymph nodes of axilla and upper limb |
| Cancer Diagnosis | ICD-10 | C81.75 | Other Hodgkin lymphoma, lymph nodes of inguinal region and lower limb |
| Cancer Diagnosis | ICD-10 | C81.76 | Other Hodgkin lymphoma, intrapelvic lymph nodes |
| Cancer Diagnosis | ICD-10 | C81.77 | Other Hodgkin lymphoma, spleen |
| Cancer Diagnosis | ICD-10 | C81.78 | Other Hodgkin lymphoma, lymph nodes of multiple sites |
| Cancer Diagnosis | ICD-10 | C81.79 | Other Hodgkin lymphoma, extranodal and solid organ sites |
| Cancer Diagnosis | ICD-10 | C81.90 | Hodgkin lymphoma, unspecified, unspecified site |
| Cancer Diagnosis | ICD-10 | C81.91 | Hodgkin lymphoma, unspecified, lymph nodes of head, face, and neck |
| Cancer Diagnosis | ICD-10 | C81.92 | Hodgkin lymphoma, unspecified, intrathoracic lymph nodes |
| Cancer Diagnosis | ICD-10 | C81.93 | Hodgkin lymphoma, unspecified, intra-abdominal lymph nodes |
| Cancer Diagnosis | ICD-10 | C81.94 | Hodgkin lymphoma, unspecified, lymph nodes of axilla and upper limb |
| Cancer Diagnosis | ICD-10 | C81.95 | Hodgkin lymphoma, unspecified, lymph nodes of inguinal region and lower limb |
| Cancer Diagnosis | ICD-10 | C81.96 | Hodgkin lymphoma, unspecified, intrapelvic lymph nodes |
| Cancer Diagnosis | ICD-10 | C81.97 | Hodgkin lymphoma, unspecified, spleen |
| Cancer Diagnosis | ICD-10 | C81.98 | Hodgkin lymphoma, unspecified, lymph nodes of multiple sites |
| Cancer Diagnosis | ICD-10 | C81.99 | Hodgkin lymphoma, unspecified, extranodal and solid organ sites |
| Cancer Diagnosis | ICD-10 | C82.00 | Follicular lymphoma grade I, unspecified site |
| Cancer Diagnosis | ICD-10 | C82.01 | Follicular lymphoma grade I, lymph nodes of head, face, and neck |
| Cancer Diagnosis | ICD-10 | C82.02 | Follicular lymphoma grade I, intrathoracic lymph nodes |
| Cancer Diagnosis | ICD-10 | C82.03 | Follicular lymphoma grade I, intra-abdominal lymph nodes |
| Cancer Diagnosis | ICD-10 | C82.04 | Follicular lymphoma grade I, lymph nodes of axilla and upper limb |
| Cancer Diagnosis | ICD-10 | C82.05 | Follicular lymphoma grade I, lymph nodes of inguinal region and lower limb |
| Cancer Diagnosis | ICD-10 | C82.06 | Follicular lymphoma grade I, intrapelvic lymph nodes |
| Cancer Diagnosis | ICD-10 | C82.07 | Follicular lymphoma grade I, spleen |
| Cancer Diagnosis | ICD-10 | C82.08 | Follicular lymphoma grade I, lymph nodes of multiple sites |
| Cancer Diagnosis | ICD-10 | C82.09 | Follicular lymphoma grade I, extranodal and solid organ sites |
| Cancer Diagnosis | ICD-10 | C82.10 | Follicular lymphoma grade II, unspecified site |
| Cancer Diagnosis | ICD-10 | C82.11 | Follicular lymphoma grade II, lymph nodes of head, face, and neck |
| Cancer Diagnosis | ICD-10 | C82.12 | Follicular lymphoma grade II, intrathoracic lymph nodes |
| Cancer Diagnosis | ICD-10 | C82.13 | Follicular lymphoma grade II, intra-abdominal lymph nodes |
| Cancer Diagnosis | ICD-10 | C82.14 | Follicular lymphoma grade II, lymph nodes of axilla and upper limb |
| Cancer Diagnosis | ICD-10 | C82.15 | Follicular lymphoma grade II, lymph nodes of inguinal region and lower limb |
| Cancer Diagnosis | ICD-10 | C82.16 | Follicular lymphoma grade II, intrapelvic lymph nodes |
| Cancer Diagnosis | ICD-10 | C82.17 | Follicular lymphoma grade II, spleen |
| Cancer Diagnosis | ICD-10 | C82.18 | Follicular lymphoma grade II, lymph nodes of multiple sites |
| Cancer Diagnosis | ICD-10 | C82.19 | Follicular lymphoma grade II, extranodal and solid organ sites |
| Cancer Diagnosis | ICD-10 | C82.20 | Follicular lymphoma grade III, unspecified, unspecified site |
| Cancer Diagnosis | ICD-10 | C82.21 | Follicular lymphoma grade III, unspecified, lymph nodes of head, face, and neck |
| Cancer Diagnosis | ICD-10 | C82.22 | Follicular lymphoma grade III, unspecified, intrathoracic lymph nodes |
| Cancer Diagnosis | ICD-10 | C82.23 | Follicular lymphoma grade III, unspecified, intra-abdominal lymph nodes |
| Cancer Diagnosis | ICD-10 | C82.24 | Follicular lymphoma grade III, unspecified, lymph nodes of axilla and upper limb |
| Cancer Diagnosis | ICD-10 | C82.25 | Follicular lymphoma grade III, unspecified, lymph nodes of inguinal region and lower limb |
| Cancer Diagnosis | ICD-10 | C82.26 | Follicular lymphoma grade III, unspecified, intrapelvic lymph nodes |
| Cancer Diagnosis | ICD-10 | C82.27 | Follicular lymphoma grade III, unspecified, spleen |
| Cancer Diagnosis | ICD-10 | C82.28 | Follicular lymphoma grade III, unspecified, lymph nodes of multiple sites |
| Cancer Diagnosis | ICD-10 | C82.29 | Follicular lymphoma grade III, unspecified, extranodal and solid organ sites |
| Cancer Diagnosis | ICD-10 | C82.30 | Follicular lymphoma grade IIIa, unspecified site |
| Cancer Diagnosis | ICD-10 | C82.31 | Follicular lymphoma grade IIIa, lymph nodes of head, face, and neck |
| Cancer Diagnosis | ICD-10 | C82.32 | Follicular lymphoma grade IIIa, intrathoracic lymph nodes |
| Cancer Diagnosis | ICD-10 | C82.33 | Follicular lymphoma grade IIIa, intra-abdominal lymph nodes |
| Cancer Diagnosis | ICD-10 | C82.34 | Follicular lymphoma grade IIIa, lymph nodes of axilla and upper limb |
| Cancer Diagnosis | ICD-10 | C82.35 | Follicular lymphoma grade IIIa, lymph nodes of inguinal region and lower limb |
| Cancer Diagnosis | ICD-10 | C82.36 | Follicular lymphoma grade IIIa, intrapelvic lymph nodes |
| Cancer Diagnosis | ICD-10 | C82.37 | Follicular lymphoma grade IIIa, spleen |
| Cancer Diagnosis | ICD-10 | C82.38 | Follicular lymphoma grade IIIa, lymph nodes of multiple sites |
| Cancer Diagnosis | ICD-10 | C82.39 | Follicular lymphoma grade IIIa, extranodal and solid organ sites |
| Cancer Diagnosis | ICD-10 | C82.40 | Follicular lymphoma grade IIIb, unspecified site |
| Cancer Diagnosis | ICD-10 | C82.41 | Follicular lymphoma grade IIIb, lymph nodes of head, face, and neck |
| Cancer Diagnosis | ICD-10 | C82.42 | Follicular lymphoma grade IIIb, intrathoracic lymph nodes |
| Cancer Diagnosis | ICD-10 | C82.43 | Follicular lymphoma grade IIIb, intra-abdominal lymph nodes |
| Cancer Diagnosis | ICD-10 | C82.44 | Follicular lymphoma grade IIIb, lymph nodes of axilla and upper limb |
| Cancer Diagnosis | ICD-10 | C82.45 | Follicular lymphoma grade IIIb, lymph nodes of inguinal region and lower limb |
| Cancer Diagnosis | ICD-10 | C82.46 | Follicular lymphoma grade IIIb, intrapelvic lymph nodes |
| Cancer Diagnosis | ICD-10 | C82.47 | Follicular lymphoma grade IIIb, spleen |
| Cancer Diagnosis | ICD-10 | C82.48 | Follicular lymphoma grade IIIb, lymph nodes of multiple sites |
| Cancer Diagnosis | ICD-10 | C82.49 | Follicular lymphoma grade IIIb, extranodal and solid organ sites |
| Cancer Diagnosis | ICD-10 | C82.50 | Diffuse follicle center lymphoma, unspecified site |
| Cancer Diagnosis | ICD-10 | C82.51 | Diffuse follicle center lymphoma, lymph nodes of head, face, and neck |
| Cancer Diagnosis | ICD-10 | C82.52 | Diffuse follicle center lymphoma, intrathoracic lymph nodes |
| Cancer Diagnosis | ICD-10 | C82.53 | Diffuse follicle center lymphoma, intra-abdominal lymph nodes |
| Cancer Diagnosis | ICD-10 | C82.54 | Diffuse follicle center lymphoma, lymph nodes of axilla and upper limb |
| Cancer Diagnosis | ICD-10 | C82.55 | Diffuse follicle center lymphoma, lymph nodes of inguinal region and lower limb |
| Cancer Diagnosis | ICD-10 | C82.56 | Diffuse follicle center lymphoma, intrapelvic lymph nodes |
| Cancer Diagnosis | ICD-10 | C82.57 | Diffuse follicle center lymphoma, spleen |
| Cancer Diagnosis | ICD-10 | C82.58 | Diffuse follicle center lymphoma, lymph nodes of multiple sites |
| Cancer Diagnosis | ICD-10 | C82.59 | Diffuse follicle center lymphoma, extranodal and solid organ sites |
| Cancer Diagnosis | ICD-10 | C82.60 | Cutaneous follicle center lymphoma, unspecified site |
| Cancer Diagnosis | ICD-10 | C82.61 | Cutaneous follicle center lymphoma, lymph nodes of head, face, and neck |
| Cancer Diagnosis | ICD-10 | C82.62 | Cutaneous follicle center lymphoma, intrathoracic lymph nodes |
| Cancer Diagnosis | ICD-10 | C82.63 | Cutaneous follicle center lymphoma, intra-abdominal lymph nodes |
| Cancer Diagnosis | ICD-10 | C82.64 | Cutaneous follicle center lymphoma, lymph nodes of axilla and upper limb |
| Cancer Diagnosis | ICD-10 | C82.65 | Cutaneous follicle center lymphoma, lymph nodes of inguinal region and lower limb |
| Cancer Diagnosis | ICD-10 | C82.66 | Cutaneous follicle center lymphoma, intrapelvic lymph nodes |
| Cancer Diagnosis | ICD-10 | C82.67 | Cutaneous follicle center lymphoma, spleen |
| Cancer Diagnosis | ICD-10 | C82.68 | Cutaneous follicle center lymphoma, lymph nodes of multiple sites |
| Cancer Diagnosis | ICD-10 | C82.69 | Cutaneous follicle center lymphoma, extranodal and solid organ sites |
| Cancer Diagnosis | ICD-10 | C82.80 | Other types of follicular lymphoma, unspecified site |
| Cancer Diagnosis | ICD-10 | C82.81 | Other types of follicular lymphoma, lymph nodes of head, face, and neck |
| Cancer Diagnosis | ICD-10 | C82.82 | Other types of follicular lymphoma, intrathoracic lymph nodes |
| Cancer Diagnosis | ICD-10 | C82.83 | Other types of follicular lymphoma, intra-abdominal lymph nodes |
| Cancer Diagnosis | ICD-10 | C82.84 | Other types of follicular lymphoma, lymph nodes of axilla and upper limb |
| Cancer Diagnosis | ICD-10 | C82.85 | Other types of follicular lymphoma, lymph nodes of inguinal region and lower limb |
| Cancer Diagnosis | ICD-10 | C82.86 | Other types of follicular lymphoma, intrapelvic lymph nodes |
| Cancer Diagnosis | ICD-10 | C82.87 | Other types of follicular lymphoma, spleen |
| Cancer Diagnosis | ICD-10 | C82.88 | Other types of follicular lymphoma, lymph nodes of multiple sites |
| Cancer Diagnosis | ICD-10 | C82.89 | Other types of follicular lymphoma, extranodal and solid organ sites |
| Cancer Diagnosis | ICD-10 | C82.90 | Follicular lymphoma, unspecified, unspecified site |
| Cancer Diagnosis | ICD-10 | C82.91 | Follicular lymphoma, unspecified, lymph nodes of head, face, and neck |
| Cancer Diagnosis | ICD-10 | C82.92 | Follicular lymphoma, unspecified, intrathoracic lymph nodes |
| Cancer Diagnosis | ICD-10 | C82.93 | Follicular lymphoma, unspecified, intra-abdominal lymph nodes |
| Cancer Diagnosis | ICD-10 | C82.94 | Follicular lymphoma, unspecified, lymph nodes of axilla and upper limb |
| Cancer Diagnosis | ICD-10 | C82.95 | Follicular lymphoma, unspecified, lymph nodes of inguinal region and lower limb |
| Cancer Diagnosis | ICD-10 | C82.96 | Follicular lymphoma, unspecified, intrapelvic lymph nodes |
| Cancer Diagnosis | ICD-10 | C82.97 | Follicular lymphoma, unspecified, spleen |
| Cancer Diagnosis | ICD-10 | C82.98 | Follicular lymphoma, unspecified, lymph nodes of multiple sites |
| Cancer Diagnosis | ICD-10 | C82.99 | Follicular lymphoma, unspecified, extranodal and solid organ sites |
| Cancer Diagnosis | ICD-10 | C83.00 | Small cell B-cell lymphoma, unspecified site |
| Cancer Diagnosis | ICD-10 | C83.01 | Small cell B-cell lymphoma, lymph nodes of head, face, and neck |
| Cancer Diagnosis | ICD-10 | C83.02 | Small cell B-cell lymphoma, intrathoracic lymph nodes |
| Cancer Diagnosis | ICD-10 | C83.03 | Small cell B-cell lymphoma, intra-abdominal lymph nodes |
| Cancer Diagnosis | ICD-10 | C83.04 | Small cell B-cell lymphoma, lymph nodes of axilla and upper limb |
| Cancer Diagnosis | ICD-10 | C83.05 | Small cell B-cell lymphoma, lymph nodes of inguinal region and lower limb |
| Cancer Diagnosis | ICD-10 | C83.06 | Small cell B-cell lymphoma, intrapelvic lymph nodes |
| Cancer Diagnosis | ICD-10 | C83.07 | Small cell B-cell lymphoma, spleen |
| Cancer Diagnosis | ICD-10 | C83.08 | Small cell B-cell lymphoma, lymph nodes of multiple sites |
| Cancer Diagnosis | ICD-10 | C83.09 | Small cell B-cell lymphoma, extranodal and solid organ sites |
| Cancer Diagnosis | ICD-10 | C83.10 | Mantle cell lymphoma, unspecified site |
| Cancer Diagnosis | ICD-10 | C83.11 | Mantle cell lymphoma, lymph nodes of head, face, and neck |
| Cancer Diagnosis | ICD-10 | C83.12 | Mantle cell lymphoma, intrathoracic lymph nodes |
| Cancer Diagnosis | ICD-10 | C83.13 | Mantle cell lymphoma, intra-abdominal lymph nodes |
| Cancer Diagnosis | ICD-10 | C83.14 | Mantle cell lymphoma, lymph nodes of axilla and upper limb |
| Cancer Diagnosis | ICD-10 | C83.15 | Mantle cell lymphoma, lymph nodes of inguinal region and lower limb |
| Cancer Diagnosis | ICD-10 | C83.16 | Mantle cell lymphoma, intrapelvic lymph nodes |
| Cancer Diagnosis | ICD-10 | C83.17 | Mantle cell lymphoma, spleen |
| Cancer Diagnosis | ICD-10 | C83.18 | Mantle cell lymphoma, lymph nodes of multiple sites |
| Cancer Diagnosis | ICD-10 | C83.19 | Mantle cell lymphoma, extranodal and solid organ sites |
| Cancer Diagnosis | ICD-10 | C83.30 | Diffuse large B-cell lymphoma, unspecified site |
| Cancer Diagnosis | ICD-10 | C83.31 | Diffuse large B-cell lymphoma, lymph nodes of head, face, and neck |
| Cancer Diagnosis | ICD-10 | C83.32 | Diffuse large B-cell lymphoma, intrathoracic lymph nodes |
| Cancer Diagnosis | ICD-10 | C83.33 | Diffuse large B-cell lymphoma, intra-abdominal lymph nodes |
| Cancer Diagnosis | ICD-10 | C83.34 | Diffuse large B-cell lymphoma, lymph nodes of axilla and upper limb |
| Cancer Diagnosis | ICD-10 | C83.35 | Diffuse large B-cell lymphoma, lymph nodes of inguinal region and lower limb |
| Cancer Diagnosis | ICD-10 | C83.36 | Diffuse large B-cell lymphoma, intrapelvic lymph nodes |
| Cancer Diagnosis | ICD-10 | C83.37 | Diffuse large B-cell lymphoma, spleen |
| Cancer Diagnosis | ICD-10 | C83.38 | Diffuse large B-cell lymphoma, lymph nodes of multiple sites |
| Cancer Diagnosis | ICD-10 | C83.39 | Diffuse large B-cell lymphoma, extranodal and solid organ sites |
| Cancer Diagnosis | ICD-10 | C83.50 | Lymphoblastic (diffuse) lymphoma, unspecified site |
| Cancer Diagnosis | ICD-10 | C83.51 | Lymphoblastic (diffuse) lymphoma, lymph nodes of head, face, and neck |
| Cancer Diagnosis | ICD-10 | C83.52 | Lymphoblastic (diffuse) lymphoma, intrathoracic lymph nodes |
| Cancer Diagnosis | ICD-10 | C83.53 | Lymphoblastic (diffuse) lymphoma, intra-abdominal lymph nodes |
| Cancer Diagnosis | ICD-10 | C83.54 | Lymphoblastic (diffuse) lymphoma, lymph nodes of axilla and upper limb |
| Cancer Diagnosis | ICD-10 | C83.55 | Lymphoblastic (diffuse) lymphoma, lymph nodes of inguinal region and lower limb |
| Cancer Diagnosis | ICD-10 | C83.56 | Lymphoblastic (diffuse) lymphoma, intrapelvic lymph nodes |
| Cancer Diagnosis | ICD-10 | C83.57 | Lymphoblastic (diffuse) lymphoma, spleen |
| Cancer Diagnosis | ICD-10 | C83.58 | Lymphoblastic (diffuse) lymphoma, lymph nodes of multiple sites |
| Cancer Diagnosis | ICD-10 | C83.59 | Lymphoblastic (diffuse) lymphoma, extranodal and solid organ sites |
| Cancer Diagnosis | ICD-10 | C83.70 | Burkitt lymphoma, unspecified site |
| Cancer Diagnosis | ICD-10 | C83.71 | Burkitt lymphoma, lymph nodes of head, face, and neck |
| Cancer Diagnosis | ICD-10 | C83.72 | Burkitt lymphoma, intrathoracic lymph nodes |
| Cancer Diagnosis | ICD-10 | C83.73 | Burkitt lymphoma, intra-abdominal lymph nodes |
| Cancer Diagnosis | ICD-10 | C83.74 | Burkitt lymphoma, lymph nodes of axilla and upper limb |
| Cancer Diagnosis | ICD-10 | C83.75 | Burkitt lymphoma, lymph nodes of inguinal region and lower limb |
| Cancer Diagnosis | ICD-10 | C83.76 | Burkitt lymphoma, intrapelvic lymph nodes |
| Cancer Diagnosis | ICD-10 | C83.77 | Burkitt lymphoma, spleen |
| Cancer Diagnosis | ICD-10 | C83.78 | Burkitt lymphoma, lymph nodes of multiple sites |
| Cancer Diagnosis | ICD-10 | C83.79 | Burkitt lymphoma, extranodal and solid organ sites |
| Cancer Diagnosis | ICD-10 | C83.80 | Other non-follicular lymphoma, unspecified site |
| Cancer Diagnosis | ICD-10 | C83.81 | Other non-follicular lymphoma, lymph nodes of head, face, and neck |
| Cancer Diagnosis | ICD-10 | C83.82 | Other non-follicular lymphoma, intrathoracic lymph nodes |
| Cancer Diagnosis | ICD-10 | C83.83 | Other non-follicular lymphoma, intra-abdominal lymph nodes |
| Cancer Diagnosis | ICD-10 | C83.84 | Other non-follicular lymphoma, lymph nodes of axilla and upper limb |
| Cancer Diagnosis | ICD-10 | C83.85 | Other non-follicular lymphoma, lymph nodes of inguinal region and lower limb |
| Cancer Diagnosis | ICD-10 | C83.86 | Other non-follicular lymphoma, intrapelvic lymph nodes |
| Cancer Diagnosis | ICD-10 | C83.87 | Other non-follicular lymphoma, spleen |
| Cancer Diagnosis | ICD-10 | C83.88 | Other non-follicular lymphoma, lymph nodes of multiple sites |
| Cancer Diagnosis | ICD-10 | C83.89 | Other non-follicular lymphoma, extranodal and solid organ sites |
| Cancer Diagnosis | ICD-10 | C83.90 | Non-follicular (diffuse) lymphoma, unspecified, unspecified site |
| Cancer Diagnosis | ICD-10 | C83.91 | Non-follicular (diffuse) lymphoma, unspecified, lymph nodes of head, face, and neck |
| Cancer Diagnosis | ICD-10 | C83.92 | Non-follicular (diffuse) lymphoma, unspecified, intrathoracic lymph nodes |
| Cancer Diagnosis | ICD-10 | C83.93 | Non-follicular (diffuse) lymphoma, unspecified, intra-abdominal lymph nodes |
| Cancer Diagnosis | ICD-10 | C83.94 | Non-follicular (diffuse) lymphoma, unspecified, lymph nodes of axilla and upper limb |
| Cancer Diagnosis | ICD-10 | C83.95 | Non-follicular (diffuse) lymphoma, unspecified, lymph nodes of inguinal region and lower limb |
| Cancer Diagnosis | ICD-10 | C83.96 | Non-follicular (diffuse) lymphoma, unspecified, intrapelvic lymph nodes |
| Cancer Diagnosis | ICD-10 | C83.97 | Non-follicular (diffuse) lymphoma, unspecified, spleen |
| Cancer Diagnosis | ICD-10 | C83.98 | Non-follicular (diffuse) lymphoma, unspecified, lymph nodes of multiple sites |
| Cancer Diagnosis | ICD-10 | C83.99 | Non-follicular (diffuse) lymphoma, unspecified, extranodal and solid organ sites |
| Cancer Diagnosis | ICD-10 | C84.00 | Mycosis fungoides, unspecified site |
| Cancer Diagnosis | ICD-10 | C84.01 | Mycosis fungoides, lymph nodes of head, face, and neck |
| Cancer Diagnosis | ICD-10 | C84.02 | Mycosis fungoides, intrathoracic lymph nodes |
| Cancer Diagnosis | ICD-10 | C84.03 | Mycosis fungoides, intra-abdominal lymph nodes |
| Cancer Diagnosis | ICD-10 | C84.04 | Mycosis fungoides, lymph nodes of axilla and upper limb |
| Cancer Diagnosis | ICD-10 | C84.05 | Mycosis fungoides, lymph nodes of inguinal region and lower limb |
| Cancer Diagnosis | ICD-10 | C84.06 | Mycosis fungoides, intrapelvic lymph nodes |
| Cancer Diagnosis | ICD-10 | C84.07 | Mycosis fungoides, spleen |
| Cancer Diagnosis | ICD-10 | C84.08 | Mycosis fungoides, lymph nodes of multiple sites |
| Cancer Diagnosis | ICD-10 | C84.09 | Mycosis fungoides, extranodal and solid organ sites |
| Cancer Diagnosis | ICD-10 | C84.10 | Sézary disease, unspecified site |
| Cancer Diagnosis | ICD-10 | C84.11 | Sézary disease, lymph nodes of head, face, and neck |
| Cancer Diagnosis | ICD-10 | C84.12 | Sézary disease, intrathoracic lymph nodes |
| Cancer Diagnosis | ICD-10 | C84.13 | Sézary disease, intra-abdominal lymph nodes |
| Cancer Diagnosis | ICD-10 | C84.14 | Sézary disease, lymph nodes of axilla and upper limb |
| Cancer Diagnosis | ICD-10 | C84.15 | Sézary disease, lymph nodes of inguinal region and lower limb |
| Cancer Diagnosis | ICD-10 | C84.16 | Sézary disease, intrapelvic lymph nodes |
| Cancer Diagnosis | ICD-10 | C84.17 | Sézary disease, spleen |
| Cancer Diagnosis | ICD-10 | C84.18 | Sézary disease, lymph nodes of multiple sites |
| Cancer Diagnosis | ICD-10 | C84.19 | Sézary disease, extranodal and solid organ sites |
| Cancer Diagnosis | ICD-10 | C84.40 | Peripheral T-cell lymphoma, not elsewhere classified, unspecified site |
| Cancer Diagnosis | ICD-10 | C84.41 | Peripheral T-cell lymphoma, not elsewhere classified, lymph nodes of head, face, and neck |
| Cancer Diagnosis | ICD-10 | C84.42 | Peripheral T-cell lymphoma, not elsewhere classified, intrathoracic lymph nodes |
| Cancer Diagnosis | ICD-10 | C84.43 | Peripheral T-cell lymphoma, not elsewhere classified, intra-abdominal lymph nodes |
| Cancer Diagnosis | ICD-10 | C84.44 | Peripheral T-cell lymphoma, not elsewhere classified, lymph nodes of axilla and upper limb |
| Cancer Diagnosis | ICD-10 | C84.45 | Peripheral T-cell lymphoma, not elsewhere classified, lymph nodes of inguinal region and lower limb |
| Cancer Diagnosis | ICD-10 | C84.46 | Peripheral T-cell lymphoma, not elsewhere classified, intrapelvic lymph nodes |
| Cancer Diagnosis | ICD-10 | C84.47 | Peripheral T-cell lymphoma, not elsewhere classified, spleen |
| Cancer Diagnosis | ICD-10 | C84.48 | Peripheral T-cell lymphoma, not elsewhere classified, lymph nodes of multiple sites |
| Cancer Diagnosis | ICD-10 | C84.49 | Peripheral T-cell lymphoma, not elsewhere classified, extranodal and solid organ sites |
| Cancer Diagnosis | ICD-10 | C84.60 | Anaplastic large cell lymphoma, ALK-positive, unspecified site |
| Cancer Diagnosis | ICD-10 | C84.61 | Anaplastic large cell lymphoma, ALK-positive, lymph nodes of head, face, and neck |
| Cancer Diagnosis | ICD-10 | C84.62 | Anaplastic large cell lymphoma, ALK-positive, intrathoracic lymph nodes |
| Cancer Diagnosis | ICD-10 | C84.63 | Anaplastic large cell lymphoma, ALK-positive, intra-abdominal lymph nodes |
| Cancer Diagnosis | ICD-10 | C84.64 | Anaplastic large cell lymphoma, ALK-positive, lymph nodes of axilla and upper limb |
| Cancer Diagnosis | ICD-10 | C84.65 | Anaplastic large cell lymphoma, ALK-positive, lymph nodes of inguinal region and lower limb |
| Cancer Diagnosis | ICD-10 | C84.66 | Anaplastic large cell lymphoma, ALK-positive, intrapelvic lymph nodes |
| Cancer Diagnosis | ICD-10 | C84.67 | Anaplastic large cell lymphoma, ALK-positive, spleen |
| Cancer Diagnosis | ICD-10 | C84.68 | Anaplastic large cell lymphoma, ALK-positive, lymph nodes of multiple sites |
| Cancer Diagnosis | ICD-10 | C84.69 | Anaplastic large cell lymphoma, ALK-positive, extranodal and solid organ sites |
| Cancer Diagnosis | ICD-10 | C84.70 | Anaplastic large cell lymphoma, ALK-negative, unspecified site |
| Cancer Diagnosis | ICD-10 | C84.71 | Anaplastic large cell lymphoma, ALK-negative, lymph nodes of head, face, and neck |
| Cancer Diagnosis | ICD-10 | C84.72 | Anaplastic large cell lymphoma, ALK-negative, intrathoracic lymph nodes |
| Cancer Diagnosis | ICD-10 | C84.73 | Anaplastic large cell lymphoma, ALK-negative, intra-abdominal lymph nodes |
| Cancer Diagnosis | ICD-10 | C84.74 | Anaplastic large cell lymphoma, ALK-negative, lymph nodes of axilla and upper limb |
| Cancer Diagnosis | ICD-10 | C84.75 | Anaplastic large cell lymphoma, ALK-negative, lymph nodes of inguinal region and lower limb |
| Cancer Diagnosis | ICD-10 | C84.76 | Anaplastic large cell lymphoma, ALK-negative, intrapelvic lymph nodes |
| Cancer Diagnosis | ICD-10 | C84.77 | Anaplastic large cell lymphoma, ALK-negative, spleen |
| Cancer Diagnosis | ICD-10 | C84.78 | Anaplastic large cell lymphoma, ALK-negative, lymph nodes of multiple sites |
| Cancer Diagnosis | ICD-10 | C84.79 | Anaplastic large cell lymphoma, ALK-negative, extranodal and solid organ sites |
| Cancer Diagnosis | ICD-10 | C84.90 | Mature T/NK-cell lymphomas, unspecified, unspecified site |
| Cancer Diagnosis | ICD-10 | C84.91 | Mature T/NK-cell lymphomas, unspecified, lymph nodes of head, face, and neck |
| Cancer Diagnosis | ICD-10 | C84.92 | Mature T/NK-cell lymphomas, unspecified, intrathoracic lymph nodes |
| Cancer Diagnosis | ICD-10 | C84.93 | Mature T/NK-cell lymphomas, unspecified, intra-abdominal lymph nodes |
| Cancer Diagnosis | ICD-10 | C84.94 | Mature T/NK-cell lymphomas, unspecified, lymph nodes of axilla and upper limb |
| Cancer Diagnosis | ICD-10 | C84.95 | Mature T/NK-cell lymphomas, unspecified, lymph nodes of inguinal region and lower limb |
| Cancer Diagnosis | ICD-10 | C84.96 | Mature T/NK-cell lymphomas, unspecified, intrapelvic lymph nodes |
| Cancer Diagnosis | ICD-10 | C84.97 | Mature T/NK-cell lymphomas, unspecified, spleen |
| Cancer Diagnosis | ICD-10 | C84.98 | Mature T/NK-cell lymphomas, unspecified, lymph nodes of multiple sites |
| Cancer Diagnosis | ICD-10 | C84.99 | Mature T/NK-cell lymphomas, unspecified, extranodal and solid organ sites |
| Cancer Diagnosis | ICD-10 | C84.A0 | Cutaneous T-cell lymphoma, unspecified, unspecified site |
| Cancer Diagnosis | ICD-10 | C84.A1 | Cutaneous T-cell lymphoma, unspecified lymph nodes of head, face, and neck |
| Cancer Diagnosis | ICD-10 | C84.A2 | Cutaneous T-cell lymphoma, unspecified, intrathoracic lymph nodes |
| Cancer Diagnosis | ICD-10 | C84.A3 | Cutaneous T-cell lymphoma, unspecified, intra-abdominal lymph nodes |
| Cancer Diagnosis | ICD-10 | C84.A4 | Cutaneous T-cell lymphoma, unspecified, lymph nodes of axilla and upper limb |
| Cancer Diagnosis | ICD-10 | C84.A5 | Cutaneous T-cell lymphoma, unspecified, lymph nodes of inguinal region and lower limb |
| Cancer Diagnosis | ICD-10 | C84.A6 | Cutaneous T-cell lymphoma, unspecified, intrapelvic lymph nodes |
| Cancer Diagnosis | ICD-10 | C84.A7 | Cutaneous T-cell lymphoma, unspecified, spleen |
| Cancer Diagnosis | ICD-10 | C84.A8 | Cutaneous T-cell lymphoma, unspecified, lymph nodes of multiple sites |
| Cancer Diagnosis | ICD-10 | C84.A9 | Cutaneous T-cell lymphoma, unspecified, extranodal and solid organ sites |
| Cancer Diagnosis | ICD-10 | C84.Z0 | Other mature T/NK-cell lymphomas, unspecified site |
| Cancer Diagnosis | ICD-10 | C84.Z1 | Other mature T/NK-cell lymphomas, lymph nodes of head, face, and neck |
| Cancer Diagnosis | ICD-10 | C84.Z2 | Other mature T/NK-cell lymphomas, intrathoracic lymph nodes |
| Cancer Diagnosis | ICD-10 | C84.Z3 | Other mature T/NK-cell lymphomas, intra-abdominal lymph nodes |
| Cancer Diagnosis | ICD-10 | C84.Z4 | Other mature T/NK-cell lymphomas, lymph nodes of axilla and upper limb |
| Cancer Diagnosis | ICD-10 | C84.Z5 | Other mature T/NK-cell lymphomas, lymph nodes of inguinal region and lower limb |
| Cancer Diagnosis | ICD-10 | C84.Z6 | Other mature T/NK-cell lymphomas, intrapelvic lymph nodes |
| Cancer Diagnosis | ICD-10 | C84.Z7 | Other mature T/NK-cell lymphomas, spleen |
| Cancer Diagnosis | ICD-10 | C84.Z8 | Other mature T/NK-cell lymphomas, lymph nodes of multiple sites |
| Cancer Diagnosis | ICD-10 | C84.Z9 | Other mature T/NK-cell lymphomas, extranodal and solid organ sites |
| Cancer Diagnosis | ICD-10 | C85.10 | Unspecified B-cell lymphoma, unspecified site |
| Cancer Diagnosis | ICD-10 | C85.11 | Unspecified B-cell lymphoma, lymph nodes of head, face, and neck |
| Non-Diabetic Neuropathies | ICD-10 | G60.0 | Hereditary motor and sensory neuropathy |
| Non-Diabetic Neuropathies | ICD-10 | G60.8 | Other hereditary and idiopathic neuropathies |
| Non-Diabetic Neuropathies | ICD-10 | G60.9 | Hereditary and idiopathic neuropathy, unspecified |
| Non-Diabetic Neuropathies | ICD-10 | G62.0 | Drug-induced polyneuropathy |
| Non-Diabetic Neuropathies | ICD-10 | G62.1 | Alcoholic polyneuropathy |
| Non-Diabetic Neuropathies | ICD-10 | G62.2 | Polyneuropathy due to other toxic agents |
| Chronic Pain Diagnosis | ICD-10 | G89.0 | Central pain syndrome |
| Chronic Pain Diagnosis | ICD-10 | G89.29 | Other chronic pain |
| Chronic Pain Diagnosis | ICD-10 | G89.4 | Chronic pain syndrome |
| Pregnancy Diagnosis | ICD-10 | Z33 | Z33 Pregnant state [Non-Specific Code] |
| Pregnancy Diagnosis | ICD-10 | Z33.1 | Pregnant state, incidental |
| Pregnancy Diagnosis | ICD-10 | Z33.2 | Encounter for elective termination of pregnancy |
| Pregnancy Diagnosis | ICD-10 | Z33.3 | Pregnant state, gestational carrier |
| Pregnancy Diagnosis | ICD-10 | Z34 | Z34 Encounter for supervision of normal pregnancy [Non-Specific Code] |
| Pregnancy Diagnosis | ICD-10 | Z34.0 | Z34.0 Encounter for supervision of normal first pregnancy [Non-Specific Code] |
| Pregnancy Diagnosis | ICD-10 | Z34.00 | Encounter for supervision of normal first pregnancy, unspecified trimester |
| Pregnancy Diagnosis | ICD-10 | Z34.01 | Encounter for supervision of normal first pregnancy, first trimester |
| Pregnancy Diagnosis | ICD-10 | Z34.02 | Encounter for supervision of normal first pregnancy, second trimester |
| Pregnancy Diagnosis | ICD-10 | Z34.03 | Encounter for supervision of normal first pregnancy, third trimester |
| Pregnancy Diagnosis | ICD-10 | Z34.8 | Z34.8 Encounter for supervision of other normal pregnancy [Non-Specific Code] |
| Pregnancy Diagnosis | ICD-10 | Z34.80 | Encounter for supervision of other normal pregnancy, unspecified trimester |
| Pregnancy Diagnosis | ICD-10 | Z34.81 | Encounter for supervision of other normal pregnancy, first trimester |
| Pregnancy Diagnosis | ICD-10 | Z34.82 | Encounter for supervision of other normal pregnancy, second trimester |
| Pregnancy Diagnosis | ICD-10 | Z34.83 | Encounter for supervision of other normal pregnancy, third trimester |
| Pregnancy Diagnosis | ICD-10 | Z34.9 | Z34.9 Encounter for supervision of normal pregnancy, unspecified [Non-Specific Code] |
| Pregnancy Diagnosis | ICD-10 | Z34.90 | Encounter for supervision of normal pregnancy, unspecified, unspecified trimester |
| Pregnancy Diagnosis | ICD-10 | Z34.91 | Encounter for supervision of normal pregnancy, unspecified, first trimester |
| Pregnancy Diagnosis | ICD-10 | Z34.92 | Encounter for supervision of normal pregnancy, unspecified, second trimester |
| Pregnancy Diagnosis | ICD-10 | Z34.93 | Encounter for supervision of normal pregnancy, unspecified, third trimester |

Table S2. Medication Use by diabetes type and DPN severity

| **Patients with Type 1 Diabetes** | | | | | |  |
| --- | --- | --- | --- | --- | --- | --- |
| N | | 88,012 | 16,194 | 4,988 | 10,821 |  |
| CGM usage (%) | 22.8% | | 20.9% | 18.6% | 15.1% | <.0001 |
| **Patients with Type 2 Diabetes** | | | | | |  |
| N | | 1,596,810 | 144,999 | 44,257 | 74,671 |  |
| Any Insulin prescription (%) | 12.8% | | 24.5% | 33.6% | 43.5% | <.0001 |
| Any Non-Insulin prescription* | 53.6% | | 57.9% | 66.2% | 69.8% | <.0001 |
| Number of Treated Days (Mean ± SD) |  | |  |  |  |  |
| Insulin | 217 ± 100 | | 222 ± 97 | 230 ± 97 | 225 ± 98 | <.0001 |
| Non-Insulin prescription(s) | 263 ± 89 | | 270 ± 86 | 277 ± 82 | 267 ± 87 | <.0001 |

Figure S1. Distribution of DPN Status by Diabetes Type

N = 1,553,824

N = 64,132

N = 131,021

N = 39,619

Total N: T1=23,937, T2=1,764,659.
**Undetermined diabetes patients were not compared due to lack of detail on which form of diabetes the patient had (5.1% of the total study population).*
